# Hydrogel-Based Bioinks for Coaxial and Triaxial Bioprinting: A Review of Material Properties, Printing Techniques, and Applications

**DOI:** 10.3390/polym17070917

**Published:** 2025-03-28

**Authors:** Alma Tamunonengiofori Banigo, Laura Nauta, Bram Zoetebier, Marcel Karperien

**Affiliations:** Department of Developmental BioEngineering, Faculty of Science and Technology and TechMed Centre, University of Twente, Drienerlolaan 5, 7522 NB Enschede, The Netherlands; a.tamunonengioforibanigo2025@outlook.com (A.T.B.); l.nauta@utwente.nl (L.N.); b.zoetebier@utwente.nl (B.Z.)

**Keywords:** tissue engineering, bioprinting, hydrogel bioinks, coaxial and triaxial bioprinting

## Abstract

Three-dimensional bioprinting technology has emerged as a rapidly advancing multidisciplinary field with significant potential for tissue engineering applications. This technology enables the formation of complex tissues and organs by utilizing hydrogels, with or without cells, as scaffolds or structural supports. Among various bioprinting methods, advanced bioprinting using coaxial and triaxial nozzles stands out as a promising technique. Coaxial bioprinting technique simultaneously deposits two material streams through a coaxial nozzle, enabling controlled formation of an outer shell and inner core construct. In contrast, triaxial bioprinting utilizes three material streams namely the outer shell, inner shell and inner core to fabricate more complex constructs. Despite the growing interest in 3D bioprinting, the development of suitable cell-laden bioinks for creating complex tissues remains unclear. To address this gap, a systematic review was conducted using the preferred reporting items for systematic reviews and meta-analyses (PRISMA) flowchart, collecting 1621 papers from various databases, including Web of Science, PUBMED, SCOPUS, and Springer Link. After careful selection, 85 research articles focusing on coaxial and triaxial bioprinting were included in the review. Specifically, 77 research articles concentrated on coaxial bioprinting and 11 focused on triaxial bioprinting, with 3 covering both techniques. The search, conducted between 1 April and 30 September 2023, had no restrictions on publication date, and no meta-analyses were carried out due to the heterogeneity of studies. The primary objective of this review is to assess and identify the most commonly occurring cell-laden bioinks critical for successful advancements in bioprinting technologies. Specifically, the review focuses on delineating the commonly explored bioinks utilized in coaxial and triaxial bioprinting approaches. It focuses on evaluating the inherent merits of these bioinks, systematically comparing them while emphasizing their classifications, essential attributes, properties, and potential limitations within the domain of tissue engineering. Additionally, the review considers the applications of these bioinks, offering comprehensive insights into their efficacy and utility in the field of bioprinting technology. Overall, this review provides a comprehensive overview of some conditions of the relevant hydrogel bioinks used for coaxial and triaxial bioprinting of tissue constructs. Future research directions aimed at advancing the field are also briefly discussed.

## 1. Introduction

**Figure 1:**
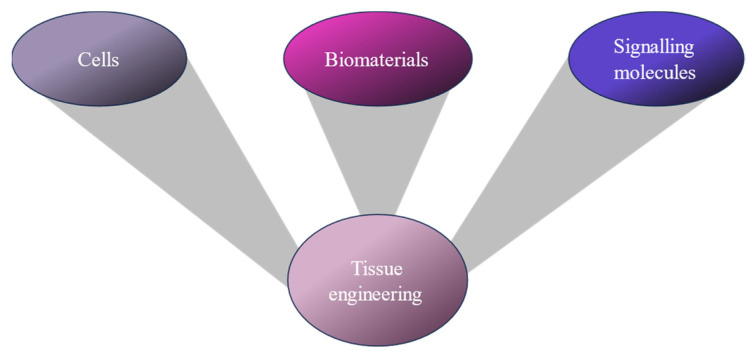
The three major components of tissue engineering.

Biofabrication is a multidisciplinary field that focuses on principles and methodologies springing from material science, biology, and engineering, converging [1] to revolutionize the field of tissue engineering and regenerative medicine (TERM). At the forefront of biofabrication is 3D bioprinting, a technology allowing the layer-by-layer deposition of three major components [biomaterials, cells, and signaling molecules (Figure 1)] to produce engineered 3D tissue constructs or models with acceptable precision and complexity [2,3,4].

### 1.1. Tissue Engineering and 3D Bioprinting Techniques

**Figure 2:**
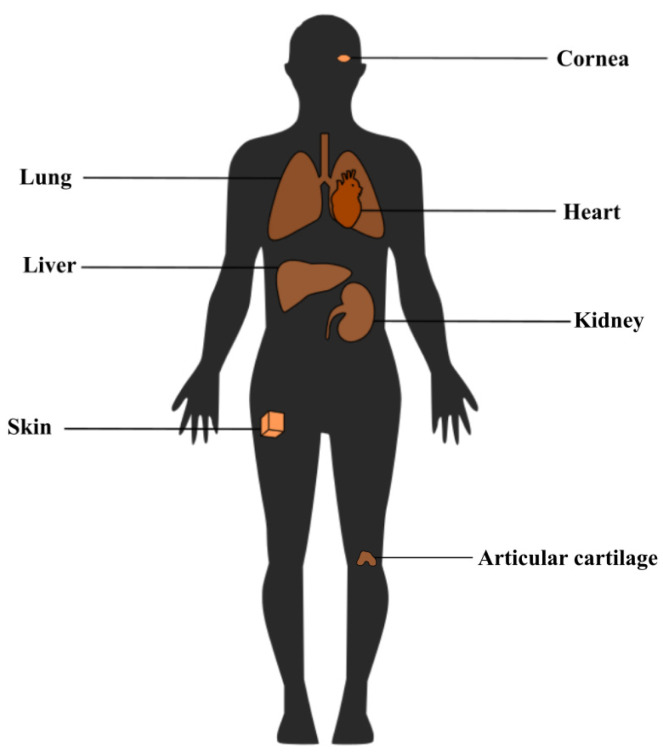
Human tissues and organs that could benefit from tissue engineering for transplantation.

**Figure 3:**
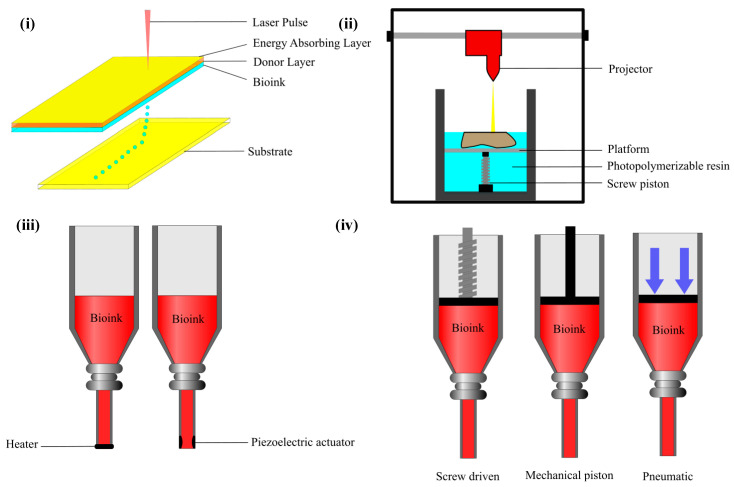
Three-dimensional bioprinting techniques: (**i**) laser-based, (**ii**) inkjet-based, (**iii**) stereolithography-based, and (**iv**) extrusion-based. Bioprinting of tissues may provide a solution for organ shortages.

**Figure 4:**
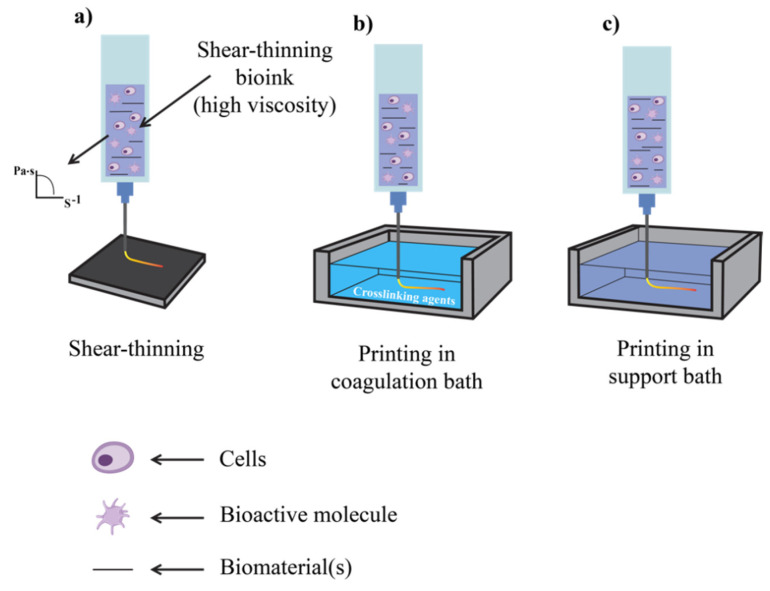
Conventional extrusion-based bioprinting conditions: (**a**) shear-thinning, (**b**) printing in a coagulation bath, and (**c**) printing in a support bath.

Tissue engineering (TE) was introduced in the 1970s with the aim of repairing and regenerating damaged tissues within the human body, marking a significant healthcare advancement (Figure 2) [5,6,7]. With the existence of 3D bioprinting, TE has overcome conventional limitations, offering the ability to design and produce complex tissue constructs that closely resemble native tissues [8] and serve as in vitro models for different purposes including disease research. Three-dimensional bioprinting is an additive manufacturing technique used in tissue engineering, where tissues and organs are created layer by layer [9,10] using various biomaterials, guided by 3D imaging and computer-aided design. The technology was first introduced by Charles Hull in 1984 with the development of stereolithography (SLA) [10]. Common 3D bioprinting techniques include laser-based, inkjet-based, stereolithography-based, and extrusion-based methods (Figure 3) [11,12,13], along with contactless and volumetric bioprinting [14,15]. A comparison of the four most common techniques (Table 1) highlights their features, including materials, printing speed, resolution, cell viability, and cell density [16,17,18,19,20,21]. Among these, extrusion-based bioprinting (EBB) is the most widely used due to its versatility, use of a range of biocompatible materials [17] with varying viscosities [16], high cell density, and viability [21]. Conventionally, EBB uses a monoaxial system with shear-thinning materials or a coagulation/support bath in specific cases (Figure 4).

Shear-thinning bioinks have viscous behavior but transition to a gel state upon deposition, facilitating extrusion during the printing period [12]. Introducing a coagulation bath can obviate the need for shear-thinning bioinks, which are essentially required in direct extrusion. The bath could contain crosslinking agents, such as calcium chloride for sodium alginate or Schiff base crosslinkers for hydrogels, like aldehyde xanthan carboxymethyl chitosan (Xan-CHO/NOCC) as examples [22,23,24]. However, nozzle tip clogging during the bioprinting process is a major issue. Alternatively, the bioink can be deposited in a granular or colloidal support bath (also termed embedded bioprinting) comprising soft micro/nano particles at a high-volume fraction. This setup facilitates the omnidirectional extrusion of cell-laden bioinks [25]. Nonetheless, drying of the bath during prolonged printing sessions is a major drawback [26]. Furthermore, constructs generated using conventional extrusion-based bioprinting may exhibit lower resolution and complexity, limited feature size, and may not be easily combinable with support materials [12,26].

While the conventional EBB bioprinting technique has demonstrated efficacy in producing tissue constructs, advanced bioprinting techniques have emerged to overcome the existing challenges (compromising between good cellular performance and shape fidelity) and to enhance the capabilities of EBB. Notably, coaxial and triaxial bioprinting techniques have garnered significant attention due to their capacity to deposit two or more bioinks simultaneously, thereby enabling the production of complex, multi-component tissue constructs with enhanced functionality [12,26].

### 1.2. Advanced Bioprinting Techniques

**Figure 5:**
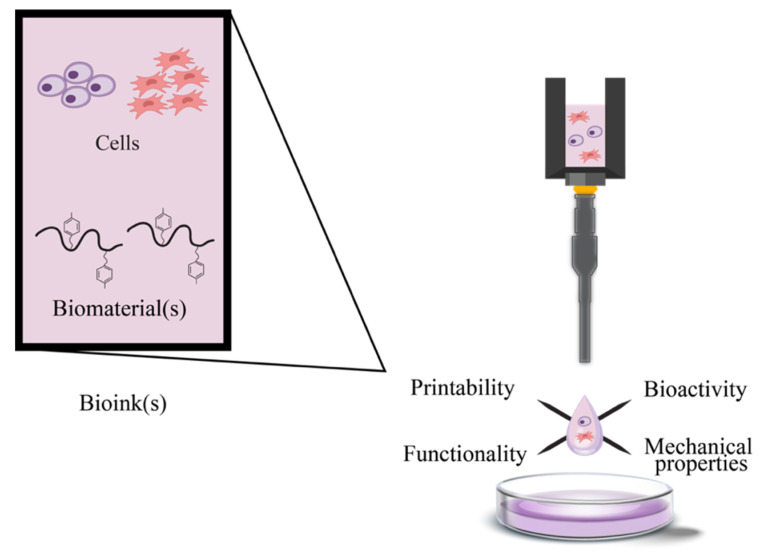
The desired features of bioinks.

**Figure 6:**
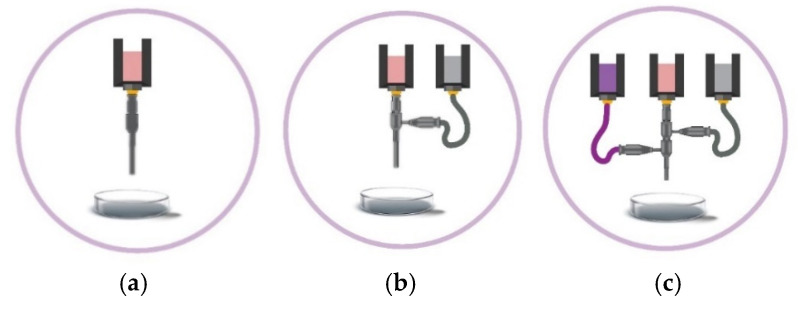
Schematic representation of nozzle types used in bioprinting: (**a**) monoaxial nozzle (**b**) coaxial nozzle (**c**) triaxial nozzle.

Coaxial [12,27,28,29,30,31,32,33] and triaxial [34,35,36,37] bioprinting techniques provide significant advancements over conventional monoaxial techniques. This is achieved by enabling more precise extrusion of bioinks (Figure 5) with a wider range of rheological properties, including viscosity, yield stress, shear recovery, and shear-thinning characteristics. This extended capability promotes the production of more complex and functional tissue constructs. The features, advantages, and limitations of advanced bioprinting techniques, such as coaxial and triaxial bioprinting, are compared to conventional monoaxial bioprinting techniques. The key components highlighted include configuration (Figure 6a–c), materials and cell types for printing, as summarized in (Table 2 and Table 3).

Coaxial [12,27,28,29,30,32] and triaxial [34,35,36,37] techniques offer enhanced control over the extrusion process. These techniques allow the creation of more complex, layered structures with varying compositions and functionalities within a single print. This capability supports the development of biomimetic tissue constructs with improved structural and functional properties compared to monoaxial techniques.

### 1.3. Bioinks

The advancement of bioprinting technologies has been significantly hindered by the absence of appropriate bioinks. Bioinks, which are typically hydrogels, consist of living cells and soft biomaterials [17] that form a cell-laden hydrogel network during the bioprinting process. These bioinks combine biomaterials with specific physiochemical properties, along with cells, to create complex 3D scaffolds through various 3D bioprinting techniques [38]. According to Whitford and Hoying, bioinks are designated as fluid materials containing cells, often supplemented with more matrix components, and are utilized in 3D printers to produce tissue-like structures [39].

For optimal performance, a bioink should possess the necessary physicochemical and bioprinting attributes. These include favorable mechanical, rheological, chemical, and biological properties, such as cell viability and proliferation. Additionally, high printing resolution is important [38,40,41,42]. These attributes should result in the following features:

(i)   The production of tissue constructs with sufficient mechanical strength and durability, while maintaining tissue-appropriate mechanics, ideally in a customizable fashion;
(ii)  Adaptable gelation and stabilization to facilitate the bioprinting of structures with precise shape fidelity;
(iii) Biocompatibility and, if required, biodegradability, resembling the natural tissue microenvironment;
(iv) The potential for chemical modifications to meet tissue-specific needs; and
(v)  The ability for large-scale production with minimal batch-to-batch variability [42,43].

In monoaxial nozzle bioprinting, bioinks comprise of a blend of biomaterials and cells, which are extruded through a monoaxial nozzle as a single-layer fiber onto a substrate [44]. Coaxial nozzle bioprinting, on the other hand, involves bioinks with multiple components, typically with cells encapsulated in a core material and surrounded by a shell material. These are deposited through a coaxial nozzle as a double-layer fiber or single-layer hollow fiber. The bioink should exhibit a viscosity ranging from 30 mPa∙s to 600 kPa∙s [45], encompassing cell signaling and attachment molecules to promote favorable cellular responses. Additionally, it must have appropriate mechanical properties for the intended application, undergo rapid gelation to maintain printing resolution, and be cytocompatible [32].

For specific applications or design requirements, cell-laden bioinks, sacrificial materials, and crosslinking agents can be used as precursors for bioprinting [35]. In triaxial nozzle bioprinting, bioinks composed of cell-laden materials, crosslinking agents, and sacrificial inks are used to produce double-layered hollow fibers, enabling the direct printing of tissue engineered vascularized constructs [35].

The design and optimization of bioinks for advanced bioprinting techniques, such as coaxial and triaxial methods, are crucial. This is particularly essential as conventional bioprinting techniques aim to engineer artificial biological and biochemical environments that promote the synchronized growth of living cells, while also possessing favorable mechanical and rheological characteristics [46]. The choice of a bioink relies on its biocompatibility, printability, stability, degradability, and intended use, ensuring no harm to cells and is compatible with the immune system [17,47]. Table 2 provides a comparison of monoaxial, coaxial, and triaxial bioprinting techniques [12,32,34,35,37,48,49,50,51,52,53], while Table 3 highlights the differences between conventional and advanced bioprinting techniques [12,31,32,33,34,35,37,53,54,55,56,57,58,59,60].

**Table 2:** Comparison among monoaxial, coaxial, and triaxial bioprinting techniques [12,32,34,35,37,48,49,50,51,52,53].

*Conditions*	*Monoaxial*	*Coaxial*	*Triaxial*
***Configuration***	A monoaxial nozzle is employed for the deposition of bioink onto a printing surface [48].	A coaxial nozzle comprising two concentric needles is utilized. The inner core and outer shell dispense bioink 1 and 2, respectively [49].	A triaxial nozzle, composed of three concentric needles, is employed. The inner core, inner shell, and outer shell dispense bioink 1, 2, and 3, respectively [35].
***Materials***	A single type of material is appropriate for monoaxial bioprinting of uniform constructs [48,50]. It should be shear-thinning. If it has a low viscosity instead, a support bath is required. It can be combined with cells and crosslinking agents within one print cartridge.	Crosslinking agents are contained in one cartridge, while hydrogels, growth factors, and cell-laden bioinks are placed in another cartridge. These components are simultaneously extruded in a coaxial manner during the printing process [12,32]. Two or more materials are considered.	This system provides increased versatility in material selection and crosslinking agents compared to other systems. It allows the simultaneous deposition of three or more materials either or not with crosslinkers, enabling precise control over the composition and properties of the printed constructs [34,35,51].
***Cells***	Cells are mixed with the material to print 3D constructs [48]. However, their performance and viability may be compromised due to shear stress during deposition and limited control over cell distribution within the printed constructs [52].	Cells are incorporated into the inner core or outer shell bioink for the printing of tissues including fibers alongside the outer shell, which serves as a protective barrier. This allows the fabrication of complex cell-laden constructs with meticulous control over cell distribution [32,53].	The cellular microenvironment shows superior control compared to the coaxial system. This allows the printing of complex constructs with many cell types [35,37].

**Table 3:** Comparison between conventional and advanced bioprinting techniques [12,31,32,33,34,35,37,53,54,55,56,57,58,59,60].

*Conditions*	*Monoaxial*	*Coaxial*	*Triaxial*
***Advantages***	It is simple to use, cost-effective, and good for printing simple structures with homogeneous bioinks.	There is precise control over concentric multi-material extrusion, enabling the use of a broader range of printable materials. Sacrificial materials can be extruded [31,32] in a single step, enhancing resolution through inline crosslinking [32]. The outer shell of a viscous bioink can act as a protective barrier for a low-viscosity inner core bioink, while regulating the inward diffusion of crosslinking agents. Banigo et al. [31,33] demonstrated that using Pluronic F-127 as a sacrificial ink, combined with hydrogen peroxide (H_2_O_2_) in the outer shell, provided temporary structural support to the inner core bioink, containing hyaluronic acid–tyramine (HA–TA) and horseradish peroxidase (HRP). This setup effectively controlled H_2_O_2_ diffusion inward, enabling stable inner core filament formation. Also, the outer shell material mitigates shear stress. This enables the printing of core/shell [54], hollow strands [55,56], and solid [53] structures, as well as continuous hollow tubes [57] and sacrificial materials [32] in a single step.	It enhances functionality by enabling simultaneous extrusion of materials from three concentric nozzles, offering greater versatility than coaxial bioprinting systems. The use of many outer and inner shell materials enables the fine-tuning of the microenvironment surrounding the printed cells [35]. This allows the printing of tissue engineered blood vessels [35,58,59] and produces tunable hollow and perfusable tubes [60], vascularized tissue-engineered bone [37], and multicellular-loaded and vascular grafts [35]. This approach overcomes limitations related to co-culturing three cell types [34], the precise loading of many cell types, and the maintenance of microfluidic channels [35].
***Disadvantages***	Maintaining high cell viability while ensuring good printability presents challenges. The shear stress and pressure exerted during the printing process can harm cells, reducing their viability [61]. Single-material prints typically have a lower resolution than coaxial or triaxial systems due to differing flow characteristics [12,32].	The design, set-up and operation complexity surpass those of the monoaxial system. Issues with ink compatibility and formulation arise, particularly in multi-material systems [32]. Simultaneously achieving the precise loading of many cell types and maintaining microfluidic channels pose challenges [35].	Designing branched or complex vascular networks, and maintaining cell viability and proliferation in high-strength hydrogels pose challenges with this system [35]. Its complexity compared to monoaxial and coaxial bioprinting systems may result in higher costs of equipment and difficulties in operating the printer.

### 1.4. Objectives

The primary objective of this review is to assess and identify most occurring cell-laden bioinks critical for successful advancements in bioprinting technologies. Specifically, the review focuses on delineating commonly explored bioinks utilized in coaxial and triaxial bioprinting approaches.

Here, it seeks to evaluate the inherent merits of these bioinks, systematically comparing them while emphasizing their classifications, essential attributes, properties, and potential limitations within the domain of tissue engineering. Additionally, the review considers the applications of these bioinks, offering comprehensive insights into their efficacy and utility in the field of bioprinting technology.

## 2. Materials and Methods

### 2.1. Collation and Identification of Manuscripts for Literature Analysis

A comprehensive review was conducted following the preferred reporting items for systematic reviews and meta-analyses (PRISMA) 2020 principles [62]. The PRISMA 2020 flow diagram allowed us to report our systematic review in a structured way by answering the following research questions:

1. What are the optimal formulations of cell-laden bioinks for advanced bioprinting technologies, particularly in coaxial and triaxial bioprinting approaches, and how do they compare in terms of their classifications, essential attributes, properties, and potential limitations within the realm of tissue engineering?
2. What are the applications of cell-laden bioinks in coaxial and triaxial bioprinting, and how do they contribute to advancements in tissue engineering?

### 2.2. Data Extraction and Analyses

Before reading these papers, many groups for extraction of data were considered. A list was prepared and classified into several groups, as follows:

i.   ***General data information:*** This section consists of the authors, publication year, article title, journal name, and publication type. These data will be used to assess the relevance and credibility of each study.
ii.  ***Biomaterials:*** This category is concentrated on the types of biomaterials used in coaxial and triaxial bioprinting. They are divided into different subgroups based on their importance and how they perform, such as their concentration and viscosity, which directly influence printability and the resulting properties of printed constructs.
iii. ***Crosslinking mechanisms:*** Some crosslinking mechanisms were reviewed, including physical, thermal, and chemical methods. The different crosslinking processes were based on the chosen type and characteristics.
iv. ***Printing parameter settings:*** The printing parameters including pressure, speed, printhead and bed temperature, and layer height were analyzed in these articles. These factors play an essential role in determining the final resolution and quality of the printed structures.
v.  ***Confirmation tests:*** Post-printing tests are important, and this section focuses on cell viability and proliferation, and more importantly, mechanical tests.

Indicatively, the materials were categorized as natural or synthetic, and also classified on the basis of the type of crosslinking (physical, thermal, or chemical), material name, cell-laden properties, or post-printing cell addition.

### 2.3. Literature Search Process

To conduct this review, we followed a structured approach with the use of a flow diagram, as outlined below [1,62].

#### 2.3.1. Identification of Studies [1,62]

Our search approach was focused on addressing the research questions, utilizing a broad range of keywords and databases. This approach was introduced to identify relevant studies concentrating on bioprintable cell-laden hydrogels for tissue engineering.

**Figure 7:**
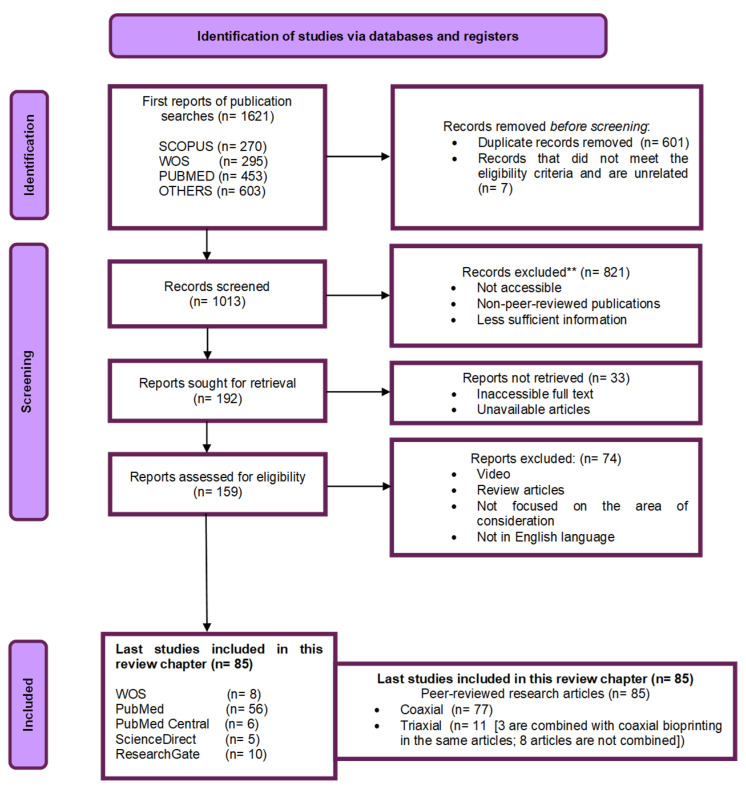
PRISMA flow chart illustrating the literature research, exclusion step, screening process, and the final included papers. Eighty-five research articles were accepted with no publication date restriction (search performed from 1 April 2023 to 30 September 2023). n in the flow chart represents total number.

In brief, the search engines made available to identify these studies are as follows: PubMed, PubMed Central, ResearchGate, ScienceDirect, Scopus, Springer, and Web of Science. The following words were used as literature search items: coaxial bioprinting, triaxial bioprinting, hydrogels (cell-laden), 3D bioprinting, tissue engineering, and tissue regeneration. Here, some records were selected and removed. They included the following:

(i)   ***Duplicate records****:* Identified through titles, author names, and publication information [62].
(ii)  ***Less relevant or unrelated records:*** Records that did not meet the eligibility criteria through a skimming method with a focus on the title, abstract, or other metadata
(iii) ***Records outside scope:*** Studies that were completely outside the scope of our review before screening (Figure 7).

In conclusion, the records removed immediately before screening were ineligible to be added in the review due to the predefined criteria.

#### 2.3.2. Screening and Selection of Studies

After removing irrelevant records, we concentrated on the remaining records due for further evaluation based on predefined inclusion and exclusion criteria [1,62]. These were categorized as follows:

(i)   ***Screened records:*** In this stage, records were reviewed by scanning through the remaining titles, abstracts, introduction (aim and objectives), methodologies, conclusions, and other metadata to select important studies.
(ii)  ***Excluded records:*** Records were excluded because they were neither accessible nor had sufficient information in the required scanned areas. Non-peer-reviewed publications, such as conference abstracts, dissertation, or case studies, were also excluded.
(iii) ***Sought for retrieval records ***[1,62]***:*** Here, we retrieved the full text of records that met the inclusion criteria based on the screening process.
(iv) ***Not retrieved records ***[1,62]***:*** Some records were not retrievable due to inaccessible full texts and unavailable articles.
(v)  ***Records assessed for eligibility ***[1]***:*** The full text of the records were retrieved and assessed for eligibility on predefined criteria. Contents including pre- to post-printing were examined to determine whether the records met the inclusion criteria in the review flow diagram (Figure 7).
(vi) ***Final excluded records:*** Records neither shown as texts (such as videos) nor written in English, among other conditions, were excluded from the final analysis. Review articles were also excluded.

### 2.4. Criteria for Inclusion

The next stage involved the selection of only original peer-reviewed papers with relevant information.

***Inclusion*** [1,62]: Researchers decided to focus on the final selected original peer-reviewed research papers with sufficient information to be included in this systematic review. The main author and first co-author carefully read the research papers in detail to extract the important data.

The search for relevant studies was performed between 1 April 2023 and 30 September 2023. No restriction was made on the publication date. No meta-analyses were carried out due to the heterogeneity of studies [1].

## 3. Results and Discussion

### 3.1. Total Research and Overall Findings

About 1621 reports were identified during the search processes (EB bioprinting of tissues for TE, coaxial, and triaxial bioprinting) in more than 5 databases (PubMed, PubMed Central, ResearchGate, ScienceDirect, Scopus, Springer, Web of Science, etc.). A total of 1013 reports were screened based on the selection conditions after removing 601 duplicates and 7 records that did not meet the eligibility criteria and were unrelated. A total of 821 were excluded from 1013 reports due to a lack of access, less sufficient information, and because they were non-peer-reviewed publications.

Then, the remaining 192 reports were sought for retrieval, out of which 33 were not retrieved. The 159 retrieved reports were eventually assessed for eligibility. A total of 74 out of 159 reports were further excluded based on various factors, including different languages (like Chinese), while many were published review articles.

Afterward, 85 published research papers about advanced bioprinting were chosen for this review chapter, of which 77 focused on coaxial and 11 on triaxial (with 3 concentrated on both coaxial and triaxial) bioprinting, as shown in Figure 7. 

**Figure 8:**
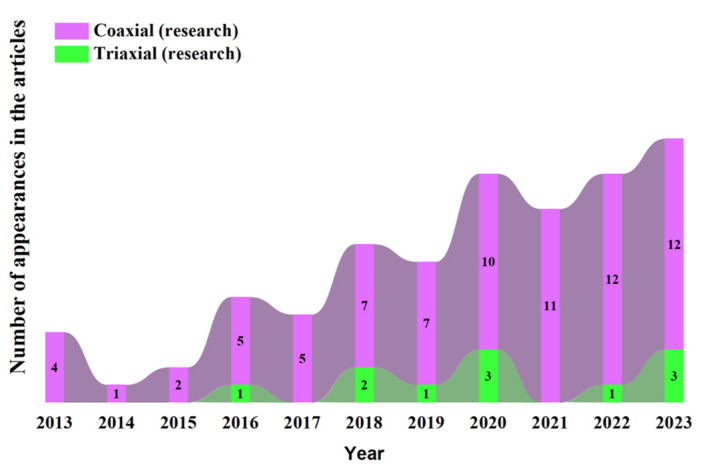
Upward trend in research articles for coaxial and triaxial bioprinting using an Origin ribbon chart (total number of appearances per year). The coaxial and triaxial bioprinting research articles are shown individually in purple and green colors, respectively.

**Figure 9:**
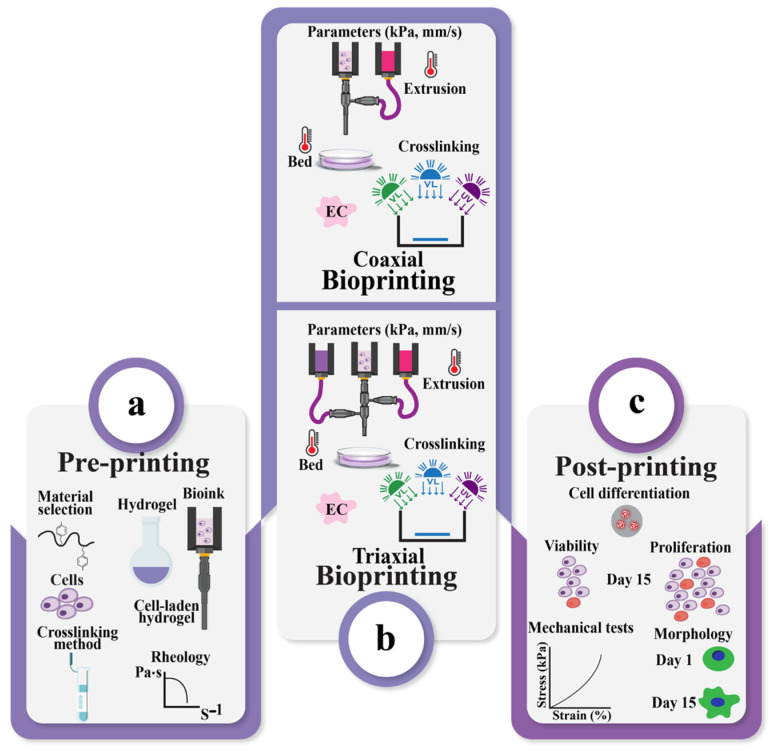
Schematic representation of three distinct bioprinting phases: (**a**) pre-printing (material and cell selection, hydrogel synthesis, and hydrogel development); (**b**) coaxial and triaxial bioprinting (parameters and crosslinking methods); (**c**) post-printing experiments (cellular studies and mechanical tests).

Following a meticulous curation process involving the selection of 85 research articles (77 exclusively dedicated to coaxial bioprinting, 11 to triaxial bioprinting, and 3 addressing both approaches) from the years 2011 to 2023, a notable trend has emerged. Over the past decade (2013–2023), there has been a discernible increase in scholarly output pertaining to both coaxial and triaxial bioprinting, characterized by a fluctuating yet upward trajectory (Figure 8). After conducting a preliminary review of the 85 research articles, it was observed that most researchers commonly segmented their research into 3 distinct phases, which we have categorized as pre-printing, coaxial or triaxial bioprinting, and post-printing phases. Notably, the selection of tests and methodologies (applied in the pre- and post-printing phases included in Figure 9) was carried out via a randomized selection.

### 3.2. Pre-Printing, Bioprinting, and Post-Printing Considerations for Hydrogel Bioinks in Coaxial and Triaxial Bioprinting

#### 3.2.1. Pre-Printing: Material Selection and Concentration

In 3D bioprinting, the selection of an appropriate bioink is crucial for effectively mimicking tissue constructs.

Bioinks, predominantly utilized in coaxial and triaxial bioprinting of both simple and complex constructs, are primarily hydrogel bioinks, as evidenced by the 85 articles reviewed [35,63,64,65]. Hydrogels are the most used bioinks due to their amenability to mild processing conditions, compatibility with various bioprinting techniques, and biocompatibility, mirroring the structural characteristics of the extracellular matrix [66,67,68]. Depending on the material’s origin, hydrogels are categorized into three main types: natural, synthetic, and natural/synthetic or hybrid hydrogels. Hydrogels, derived from either natural or synthetic sources, belong to a class of materials known for their ability to absorb and retain large volumes of water [17].

Crosslinking is a pivotal process in hydrogel gelation to ensure the structural integrity of bioprinted constructs over time. Crosslinking can be achieved through physical or chemical means [65]. Physical crosslinking involves hydrophobic interactions, hydrogen bonding, stereo complexation, electrostatic interactions, and guest–host interactions, while chemical crosslinking utilizes free radical polymerization, enzymatic reactions, condensation reactions, high-energy irradiation, or Schiff’s base reactions [69,70]. Coaxial bioprinting often employs ionic crosslinking due to its rapid crosslinking kinetics [12].

**Figure 10:**
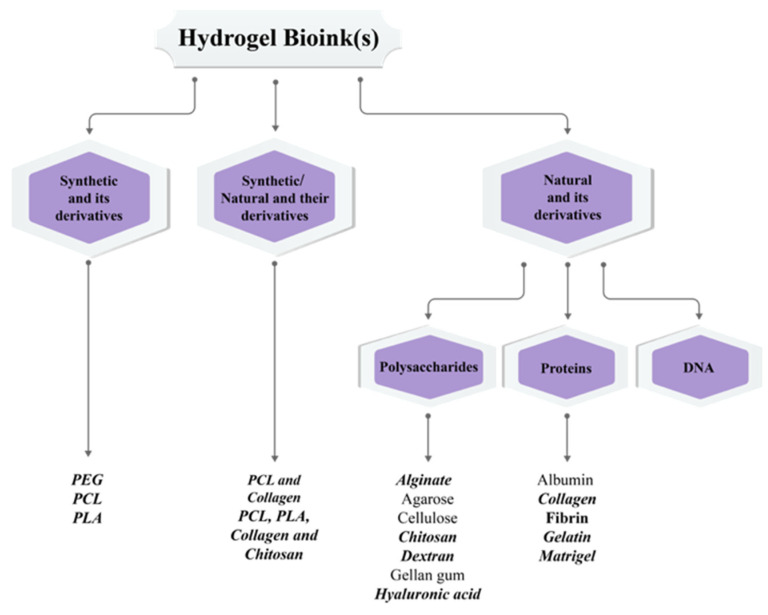
Commonly used hydrogels for cell-based 3D bioprinting. Polymers without bold font have been used for 3D bioprinting, whereas polymers in bold and italics have been explored in coaxial and/or triaxial bioprinting [71].

Several bioinks (Figure 10) extensively explored in 3D bioprinting include but are not limited to alginate, chitosan, collagen, dextran, fibrin, gelatin, hyaluronic acid {HA}, silk fibroin, Pluronic F-127, poly(ethylene) glycol {PEG} [12,17,70], poly (ε-caprolactone) {PCL} and poly (lactic acid) {PLA}. Among them, alginate, gelatin, HA, PEG, and their composites are the most frequently used hydrogel bioinks in coaxial and triaxial bioprinting, as evidenced by the 85 articles reviewed (Table 4). These bioinks were employed either independently or in conjunction with another bioink. Pluronic F-127 has primarily served more readily as a sacrificial ink to stabilize the print before crosslinking is complete rather than a bioink in coaxial and triaxial bioprinting methods. Detailed descriptions of these bioinks and their applications in coaxial and triaxial bioprinting are provided in subsequent sections.

**Table 4:** The most relevant natural, synthetic, and hybrid (natural/synthetic) bioinks explored for coaxial and triaxial bioprinting. These bioinks were utilized alone or in combination with another bioink. Pluronic F-127 has predominantly been utilized as a sacrificial ink rather than used as a bioink for printing of cells in coaxial and triaxial bioprinting techniques (2013–2023).

Types	Biopolymers	Number of Articles	Coaxial	Triaxial	Printability
**Natural**	Alginate	70	Yes	Yes	Good for extrusion
	Gelatin	23	Yes	Yes	Moderate printability
	Collagen	14	Yes	Yes	Good for biocompatible structures
	Chitosan	4	Yes	Yes	Moderate printability
	Matrigel	4	Yes	No	Low printability
	Methyl cellulose	4	Yes	Yes	Moderate printability
	dECM	2	Yes	Yes	Low printability
**Naturally derived**	Gel–MA	29	Yes	Yes	High printability for coaxial systems
	HA–MA	4	Yes	No	Moderate printability
	Gel–TA	1	Yes	No	Low printability
	HA–TA	None yet	No	No	None
**Synthetic and its derivatives**	Pluronic F-127	9	Yes	Yes	Suitable for coaxial systems
	Poly (ε-caprolactone)	4	Yes	No	High printability
	Poly (ethylene) glycol	3	Yes	Yes	Moderate printability
	PEG–DA	4	Yes	Yes	High printability

***Alginate***

**Figure 11:**
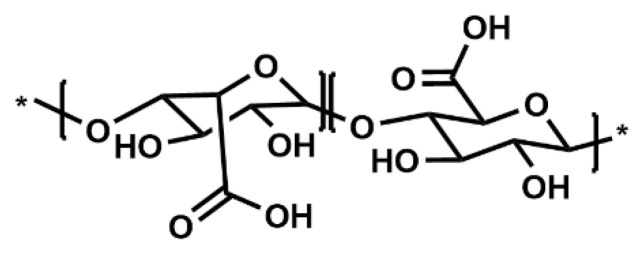
Chemical structure of alginate.

Alginate (Figure 11) stands out as a popularly known natural biopolymer for hydrogel formation in coaxial bioprinting, primarily due to its affordability, rapid ionic crosslinking capabilities, adjustable gelation kinetics, and biocompatibility [72,73,74]. The physical crosslinking of alginate with divalent cations enables gelation, a mechanism extensively harnessed in bioprinting applications [75]. The most frequently used range of alginate concentration is between 2 and 4% *w*/*v* (Table 5), because this range gives good viscosities suitable for monoaxial [1,76] and coaxial bioprinting. These concentrations provide a balance between viscosity and flowability, allowing for precise extrusion through the nozzle while maintaining structural integrity during printing. Concentrations of 2 to 4% *w*/*v* allow for efficient crosslinking and gelation upon contact with the calcium-rich crosslinking solution, ensuring the stability and shape fidelity of the printed constructs. They are preferred to minimize shear stress on encapsulated cells during printing.

Alginate hydrogels act as semi-permeable barriers, allowing for the diffusion of nutrients, oxygen, and waste products within the printed constructs. Concentrations between 2 and 4% *w*/*v* optimize the diffusion properties necessary for cell survival and function. Various groups have pioneered the development of coaxial bioprinting techniques [77,78,79,80,81,82,83], utilizing alginate for its swift crosslinking and elastic properties. Initially, tubular vascular constructs and microfluidic channels were bioprinted, both as standalone structures and within bulk gel constructs [12]. Recent advancements include the fabrication of vascular constructs containing human umbilical vein endothelial cells (HUVECs) and human umbilical vein smooth muscle cells via a four-layer coaxial nozzle arrangement with alginate bioink as the shell, resulting in improved mechanical strength with lower elasticity in four-layer constructs as compared to single-layer constructs. The produced constructs permitted cell viability and proliferation after perfusion [12,84]. Additionally, alginate-based microfibers mimicking the glioma microenvironment showed promise for drug screening studies [85].

Despite its widespread use, alginate has limitations including slow degradation, and lack of cell adhesion and proliferation. Over time, the disappearance of ionic crosslinks in alginate constructs leads to structural collapse. Also, the constraint of alginate bioink concentration to 4% *w*/*v* may impede the rate of diffusion, subsequently affecting the transportation of essential nutrients, oxygen, and metabolic byproducts across the alginate layer [86]. The higher concentrations (>4% *w*/*v*) may lead to increased shear forces, potentially compromising cell viability and functionality. More so, concentrations of alginate below 2% *w*/*v* are typically not used for coaxial and triaxial bioprinting due to several limitations, including the significant deterioration of the elastic modulus and structural integrity of the hydrogel constructs over time. Consequently, the utilization of low concentration of alginate (<2% *w*/*v*) in constructs yields soft hydrogels that compromise shape fidelity. Moreover, bioprinted structures may collapse if multilayers are formed. This impacts shape retention and resilience during handling and subsequent post-printing procedures. Also, the lack of viscosity may hinder precise deposition of the bioink and results in the shape distortion or collapse of the printed structures. They may not also adequately support cell encapsulation within the bioink, leading to poor cell viability and functionality. Alginate gels (≥4% *w*/*v*) are usually highly stiff and rigid. They entrap cells by preventing them from interacting with and responding to their mechanical environment.

**Figure 12:**
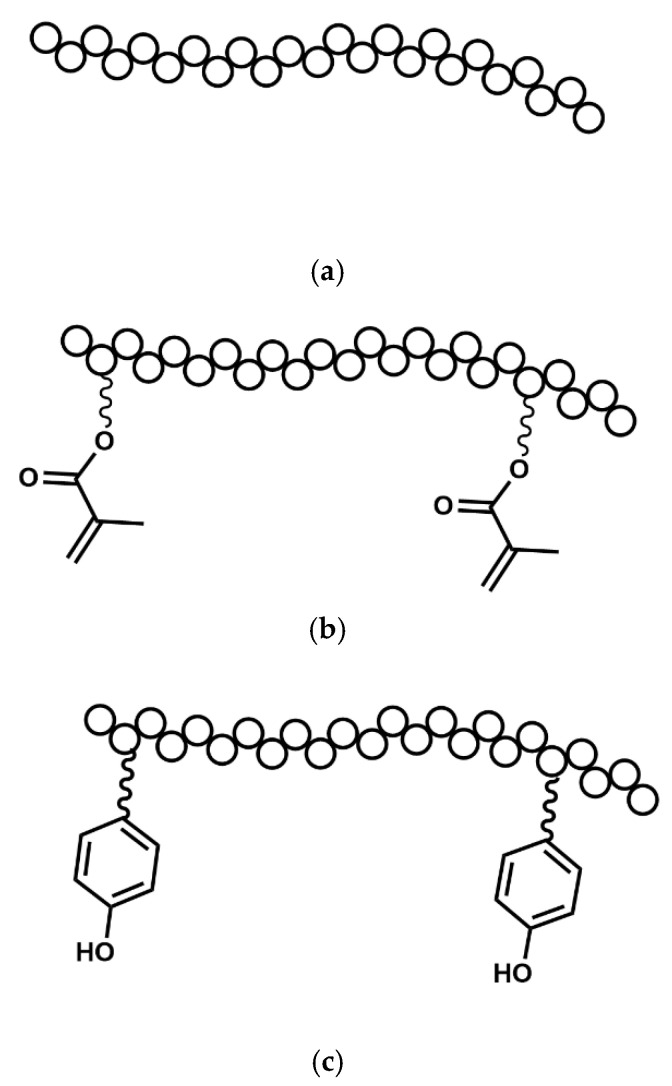
Chemical structures of: (**a**) gelatin, (**b**) Gel–MA, and (**c**) Gel–TA (circles represent individual amino acids).

To address these drawbacks, researchers have explored blending alginate with other bioinks, such as gelatin, gelatin–methacryloyl (Gel–MA), or any other suitable hydrogel, to enhance its biological properties (Figure 12a–c).

***Gelatin***

Gelatin (Figure 12a) is another well-known natural biopolymer. It is derived from the hydrolysis of collagen and exhibits a gelling temperature ranging below 20 °C to 30 °C [87] (Table 5).

This biopolymer is cytocompatible and constitutes similarities with the ECM in mammalian tissue [68]. The hydrophilic properties, chemical resilience, and biodegradable nature of gelatin hydrogels make them highly suitable as adaptable frameworks for fabricating biosynthetic tissue constructs via 3D printing techniques employing suitable cellular components [65]. Gelatin exhibits two notable characteristics, namely cellular adhesion, primarily attributed to the presence of RGD (arginine–glycine–aspartate) sequences, and thermo-responsive properties, which support its application as a supporting material [1], sacrificial ink, or printing bath [87]. The mostly used concentration range of gelatin in combination with other materials, such as alginate and gel–MA (Figure 12b), for coaxial and triaxial bioprinting is 5–10% *w*/*v*, probably due to its good thermo-responsiveness, rapid gelation, and maintenance of shape fidelity after printing, but the right concentration is highly dependent on the bioink application [1]. Incorporating chemically cross-linkable side groups, like methacrylate or methacrylamide groups, into naturally derived hydrogels, such as gelatin (such as in the case of Gel–MA) enables chemical crosslinking, thereby broadening the range of applications for these materials [65] with advantages (Table 6a–c). Gel–MA’s concentration range of 5–10% *w*/*v* (Table 5) is widely used for coaxial bioprinting due to its favorable mechanical and rheological properties. This concentration range provides a balance between viscosity and printability, allowing for precise extrusion and layer-by-layer deposition of bioink. Also, Gel–MA at 5–10% *w*/*v* shows appropriate crosslinking behaviour, ensuring structural integrity and stability of the printed constructs post-printing. Concentrations below 5% *w*/*v* may result in bioinks with insufficient viscosity, leading to poor shape fidelity and reduced structural support during printing. Conversely, concentrations exceeding 10% *w*/*v* may yield bioinks that are excessively viscous, impeding extrusion and compromising the resolution of printed structures. Moreover, higher concentrations of Gel–MA may contribute to increased stiffness of the printed constructs, which can potentially hinder cellular activity and tissue integration. Gel–MA has widely been used in 3D bioprinting and tissue engineering [60], specifically as a single bioink or mixed with other bioinks for monoaxial [88,89], coaxial [57,90,91], and triaxial [35,92,93] bioprinting of 3D constructs. Gelatin (Gel) also possesses the potential for modification with other functional groups, like tyramine (TA), thus rendering a polymer conjugate that can cross link using enzymatic reactions or visible light. Notably, Gel–TA (Figure 12c) has been introduced into coaxial bioprinting setups for the first time, albeit through the incorporation of PEG as a spacer (Gel–PEG–TA) [64]. While Gel–TA has been employed in a coaxial bioprinting setup on one occasion, its incorporation into triaxial bioprinting systems remains unexplored to date. However, comprehensive information regarding the optimal concentration in % *w*/*v* for Gel–TA in such applications is currently lacking nor how Gel–TA compares with Gel–MA.

***Hyaluronic acid (HA)*** 

**Figure 13:**
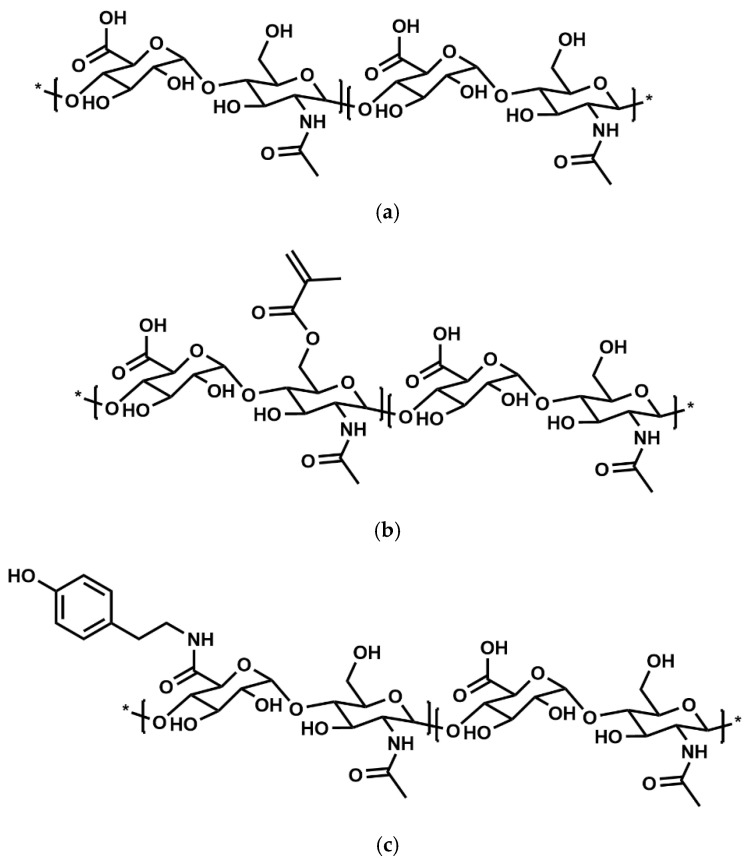
Chemical structures of: (**a**) HA, (**b**) HA–MA, and (**c**) HA–TA.

HA (Figure 13a) consists of repeating disaccharide units of D-glucuronic acid and N-acetyl-D-glucosamine, with molecular weights typically ranging between 10^5^ and 10^7^ Da, depending on its tissue source. Its extensive clinical use over the past four decades underscores its pivotal role as a naturally derived polymer in the medical field [87]. Pure and unmodified hyaluronic acid (HA) at operational concentrations does not lend itself to the production of printable bioinks due to its inherent characteristics and has limited ability to form a gel (Table 5). In the area of stabilizing hydrogels through crosslinking, hyaluronic acid (HA) can be used either in its unmodified form or as a modified derivative. The intention to modify HA depends on the specific requirements of the hydrogel system and desired properties. While modification can enhance crosslinking efficiency and control gelation kinetics, unmodified HA also demonstrates utility in some applications. Therefore, the choice between modified and unmodified HA is contingent upon the intended functionality and performance of the hydrogel.

The naturally occurring glycosaminoglycan hyaluronic acid (HA) has garnered significant attention for its application in extrusion-based 3D printing, owing to its ubiquitous presence in the human body, cytocompatibility, and versatile biological properties, coupled with its amenability to chemical modification [87].

Efforts to develop printable bioinks have increasingly focused on combining pristine HA, HA derivatives, and a range of natural and synthetic polymers. HA samples with molecular weights between 120 to 2500 kDa and concentrations ranging from 0.1 to 4% *w*/*v* have been utilized for 3D printing, particularly monoaxial [48,50,87,94] and coaxial bioprinting [31,33,65,95], enabling the fabrication of constructs with diverse mechanical properties and biodegradation rates.

While HA and its derivatives have been utilized in a monoaxial bioprinting configuration in some studies, its integration into coaxial bioprinting systems with concentrations between 0.1 and 4% *w*/*v* remains inadequately explored. Furthermore, its consideration in triaxial bioprinting setups has not yet been addressed. As a result, there is currently a dearth of comprehensive information regarding the optimal concentration, expressed as a percentage by weight (% *w*/*v*), for HA or its modified form, hyaluronic acid–methacrylate (HA–MA) and hyaluronic acid–tyramine (HA–TA), in such bioprinting applications. HA solutions in water exhibit high viscosity and shear-thinning behaviour, with a predominant viscous modulus, indicating a lack of yield stress and shape retention post-printing [87]. Consequently, HA alone is typically unsuitable for withstanding the rigors of the printing process. Instead, it is often combined with other biomaterials, i.e., either natural or synthetic polymers. HA-based bioinks can be categorized based on various compositions, as follows: (a) bioinks where HA serves as the primary component and forms a standalone material, potentially as single or multiple chemical derivatives, with properties, such as shear-thinning and post-printing crosslinking for shape preservation; (b) formulations combining HA derivatives with either natural or synthetic polymers; (c) compositions incorporating pristine or modified HA to enhance ink viscosity, final mechanical stability, and biological characteristics; and (d) combinations with mechanically robust support materials, like PCL [87].

Adding chemically cross-linkable side groups, such as methacrylate or methacrylamide groups, into hyaluronic acid (HA–MA) also allows chemical crosslinking, thereby widening the application range for these materials, especially in the monoaxial bioprinting [87,96] of 3D constructs. Due to its distinctive characteristics (Table 6a,b), the combination of HA–MA (Figure 13b) with Gel–MA has been employed for monoaxial [87,97], colinear and coaxial bioprinting [65,95,98] of tissue constructs. HA–MA/Gel–MA have not yet been explored for triaxial bioprinting.

Hyaluronic acid (HA) can also be chemically modified with functional groups, such as tyramine (TA), enabling alternative crosslinking strategies, including enzymatic approaches using horseradish peroxidase (HRP) and hydrogen peroxide (H_2_O_2_), instead of conventional UV light methods. While HA has been applied in monoaxial bioprinting systems [48,87,94], its integration into coaxial [31,33] and triaxial setups remains relatively underexplored.

Banigo et al. [31,33] were the first researchers to utilize high molecular weight hyaluronic acid–tyramine (HMW HA–TA) and enzymatic crosslinking mechanisms (HRP and H_2_O_2_) for coaxial bioprinting. In their setup, Pluronic F-127 and hydrogen peroxide (H_2_O_2_) were introduced through the outer shell (OS), while HA–TA (Figure 13c) and horseradish peroxidase (HRP) were delivered through the inner core (IC). This innovative configuration enabled near-instantaneous enzymatic crosslinking, resulting in the successful formation of core filaments containing live cells. With optimized parameters, these cell-laden filaments exhibited good cell viability, serving as a proof of concept [31,33]. This work marked the first exploration of this unique combination of materials, enzymatic crosslinking agents, and coaxial bioprinting technology for filament production.

***Poly (ethylene)*** ***glycol*** ***(PEG)***

In contemporary medical applications, biocompatible polymers play a pivotal role, with polyethylene glycol (PEG) emerging as a prominent choice.

As a synthetic polyether, PEG (Table 6c) is favored for its advantageous biological, chemical, and physical properties, including high biocompatibility, hydrophilicity, flexibility, and solubility. While it exhibits solubility in water and various solvents, it lacks degradability. Notably, crosslinking processes are commonly employed in harnessing these attributes for tailored applications.

However, through incorporation with polymers, like poly (lactic acid) {PLA} and poly (ε-caprolactone) {PCL}, amphiphilic structures can be achieved. Despite its bio-inertness, which hinders cell adhesion, proliferation, and protein adsorption, this limitation can be addressed by supplementing it with cell-binding materials to enhance cell adhesion. PEG’s amendable properties, along with its non-toxic and non-immunogenic nature, position it as a promising candidate for a wide array of biomedical applications [99].

**Figure 14:**
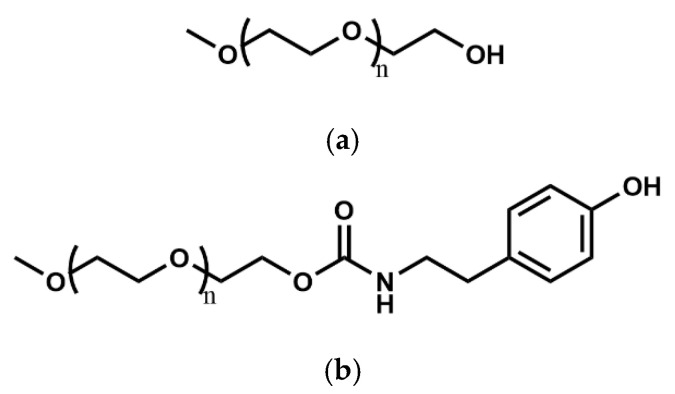
Chemical structures of: (**a**) PEG and (**b**) PEG–TA.

PEG (Figure 14a) exhibits the capacity for modification through the introduction of additional groups, such as tyramine (TA) [100], diacrylate (DA) [57]^,^ and others [101], thereby enhancing its utility in the 3D bioprinting (mono [101], coaxial [57,64,100,102], and triaxial [60]) of tissue constructs.

Although PEG and its derivatives have been employed in monoaxial bioprinting setups in certain instances, their incorporation into coaxial and triaxial bioprinting systems with concentrations below 10% *w*/*v* remains insufficiently investigated. Consequently, there is presently a lack of comprehensive data concerning the ideal concentration, quantified as (% *w*/*v*), for PEG or its modified variants including PEG–TA (Figure 14b) and PEG–DA, in these bioprinting methodologies.

***Pluronic F-127***

**Figure 15:**
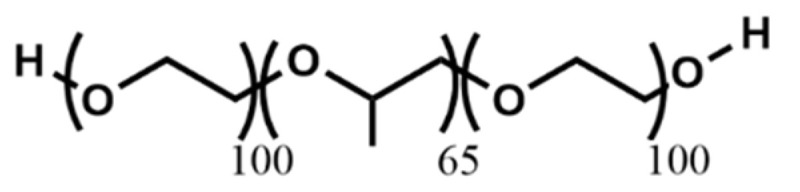
Chemical structure of Pluronic F-127.

Pluronic F-127 (poloxamer 407) is a synthetic hydrogel comprising amphiphilic copolymers containing units of polyethylene oxide (PEO) and polypropylene oxide (PPO) [103]. Pluronic F-127 (Table 6c) exhibits injectability and undergoes reversible gelation, rendering it suitable for biomedical applications [104]. Pluronic F-127 (Figure 15) is an excellent material for printability and is popularly used as a sacrificial ink for many applications, particularly in the fabrication of tubular constructs [105,106,107] and filaments [31,33] based on its thermo-reversibility. This material is extensively employed in 3D bioprinting as both a bioink and a support bath for printing tissue engineering scaffolds with embedded cells. Its thermosensitive nature enables it to encapsulate cells and to promote initial cell adhesion within defect sites due to its ability to hold cells in its structure [103].

Studies have demonstrated that it enhances cell attachment, collagen formation, and angiogenesis, suggesting its potential for tissue regeneration in poorly vascularized tissues [103].

Its optimized cell distribution in the liquid state, ease of manipulation in the solid state, and reversable thermosensitive properties make it a promising biomaterial for regenerative medicine and dentistry [103,108]. As a bioink, its ability to form a hydrogel at physiological temperatures facilitates the deposition of cells within a stable matrix, ensuring their viability and proliferation during the printing process. Additionally, the shear-thinning characteristics of Pluronic F-127 enable precise extrusion through printing nozzles, facilitating the fabrication of intricate tissue structures with high resolution. Furthermore, Pluronic F-127 is commonly utilized as a support bath or sacrificial material in monoaxial [109] and advanced (coaxial [31,33,102,107,110,111,112] and triaxial [51]) bioprinting applications.

The thermosensitive gelation properties of Pluronic F-127 enable the formation of a temporary scaffold around printed structures, offering mechanical support during printing and facilitating the subsequent removal of the scaffold through temperature-induced sol–gel transitions. This approach not only allows printing with hydrogels characterized by relatively long gelation times but also permits the printing of constructs necessitating the separation of hydrogels from crosslinkers. Consequently, it facilitates the fabrication of intricate 3D tissue constructs featuring interconnected channels or void spaces, thereby resembling the architecture of native tissues and enhancing nutrient diffusion and waste removal within the engineered constructs.

The concentration range of 15–30% *w*/*v* [31,33,51,102,107,110,113,114] for Pluronic F-127 is considered optimal for monoaxial and coaxial bioprinting, and possibly triaxial bioprinting owing to its distinctive rheological and thermosensitive characteristics.

Gioffredi et al. explored the use of 25% *w*/*v* Pluronic F-127 for monoaxial bioprinting due to its rapid gelation under physiological conditions, appropriate viscoelastic properties, and swift viscosity recuperation post-shearing [114]. This concentration allows Pluronic F-127 to maintain suitable viscosity and gelation characteristics, necessary for both supporting the printing process and ensuring structural integrity post-printing.

Banigo et al. demonstrated that 27.5% *w*/*v* Pluronic F-127, optimized for coaxial bioprinting, provided the best results when explored to print core filaments. This concentration, combined with other optimized materials and parameters, enabled successful printing with good cell viability and controlled inward diffusion of H_2_O_2_ [31,33]. However, it is suspected that concentrations below or above 27.5% *w*/*v*, could negatively hamper on the viability of cells and the printing process.

Pluronic F-127 concentrations below 15% *w*/*v* have been investigated for bioprinting but remain relatively understudied, particularly in both coaxial and triaxial bioprinting. For example, a 13% *w*/*v* Pluronic F-127 formulation mixed with CaCl_2_ in the inner core facilitated crosslinking of alginate/silk fibroin in the outer shell, enabling coaxial bioprinting of perfusable hierarchical microchannels featuring an alginate/silk fibroin double-crosslinked network [112]. This approach was employed for coaxial bioprinting of perfusable hierarchical microchannels featuring an alginate/silk fibroin double-crosslinked network [112].

On the other hand, concentrations surpassing 30% *w*/*v* have received limited attention in coaxial and triaxial bioprinting, though some studies have explored their potential under specific conditions. Gao et al. utilized a 40% *w*/*v* Pluronic F-127 solution dissolved in CaCl_2_ for coaxial cell printing, fabricating freestanding, perfusable, functional in vitro vascular models [111] and for triple-coaxial cell printing vascular grafts when combined with alginate [59]. Table 5 presents some conditions for the most relevant natural, synthetic, and hybrid (natural/synthetic) bioinks explored for coaxial and triaxial bioprinting [51,53,55,57,59,64,65,77,81,82,86,91,93,95,98,100,102,107,110,111,112,115,116,117,118,119,120,121]. Table 6a lists the pros and cons of the most relevant natural bioinks and their derivatives explored for coaxial and triaxial bioprinting [60,63,65,72,87,90,97,119,122,123,124,125,126,127,128,129,130,131,132,133,134].

Table 6b highlights the pros and cons of the most relevant natural bioinks (hybrid) and their derivatives explored for coaxial and triaxial bioprinting [60,86,95,119,132,135,136,137,138]. Table 6c summarizes the pros and cons of the most relevant synthetic and hybrid (natural/synthetic) bioinks and their derivatives explored for coaxial and triaxial bioprinting [100,103,110,139,140,141,142,143,144,145,146,147,148].

**Figure 16:**
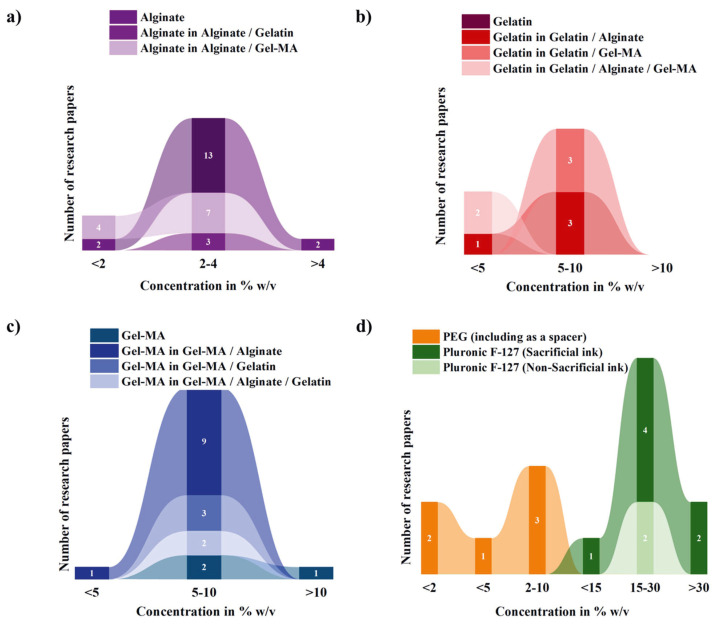
The concentration of the most commonly used materials for coaxial and triaxial bioprinting: (**a**) Alginate and its combinations. (**b**) Gelatin and its combinations. (**c**) Gel–MA and its combinations. (**d**) PEG with its derivatives and Pluronic F-127.

The concentration ranges for the biopolymers mentioned above and their combinations are shown in Figure 16a–d.

**Table 5:** Some conditions of the most relevant natural, synthetic, and hybrid (natural/synthetic) bioinks explored for coaxial and triaxial bioprinting between 2013 and 2023 [51,53,55,57,59,64,65,77,81,82,86,91,93,95,98,100,102,107,110,111,112,115,116,117,118,119,120,121].

Types	Biopolymers	Concentration (% *w*/*v*) Most Commonly Used	Gelation Method
**Natural**	Alginate [55,77,81,82,102,112,115,116]	2–4	Ionic [119] (using divalent cations, such as calcium ions)
	Gelatin [57,64,91,117,118]	5–10	Thermal [119]
	Hyaluronic acid		Shear-thinning properties
**Naturally derived**	Gel–MA [53,57,91,102,120]	5–10	Thermal/photo (ultraviolet) [86,119] (VA- 086 (2,2′-Azobis [2-methyl-N-(2 hydroxyethyl) propionamide] [95]
	Gel–TA [64]		Enzymatic (horseradish peroxidase {HRP} and hydrogen peroxide {H_2_O_2_}) [64]
	HA–MA [65,95,98]		Photo (ultraviolet) (VA- 086 (2,2′-Azobis [2-methyl-N-(2 hydroxyethyl) propionamide] [95]
	HA–TA		Shear-thinning, enzymatic, or light-based
**Synthetic and Derivatives**	PEG and its derivatives [57,93,100,121]		Chemical
	Pluronic F-127 [51,59,107,110,111,112]	15–30	Thermo-reversible

**Table 6:** (**a**) The pros and cons of the most relevant natural bioinks and their derivatives explored for coaxial and triaxial bioprinting [60,63,65,72,87,90,97,119,122,123,124,125,126,127,128,129,130,131,132,133,134]. (**b**) The pros and cons of the most relevant natural bioinks (hybrid) and their derivatives explored for coaxial and triaxial bioprinting [60,86,95,119,132,135,136,137,138]. (**c**) The pros and cons of the most relevant synthetic and hybrid (natural/synthetic) bioinks and their derivatives explored for coaxial and triaxial bioprinting [100,103,110,139,140,141,142,143,144,145,146,147,148].

(**a**)			
***Types***	**Bioinks**	**Advantages**	**Disadvantages**
***Natural bioinks***	**Alginate**	Biocompatible and cost-effective. Has good mechanical properties, structural integrity, printing resolution, shape fidelity, permeability and rapid ionic crosslinking capacity [60,63,122].	Poor cell recognition, slow degradation, bioinert [60], and limited cell adhesion or proliferation [72].
	**Gelatin**	Biocompatible, biodegradable, and water soluble [119]. Good structural stability and porosity. Has intrinsic bioactive qualities [123]. Good cell adhesion, viability and proliferation [65].	Shows poor printability based on the inherent low mechanical properties at lower concentrations, causing mechanical instability in constructs [123]. Lacks shape fidelity. Has low viscosity at low concentration. Unstable at body temperature [119].
	**HA**	Biocompatible and biodegradable [124]. Improves the viscosity of ink formulations [125,126], as well as the final mechanical stability [97] and the biological properties [127] of the printed constructs based on its biological and viscoelastic properties [87].	Pristine HA cannot form hydrogels alone [87]. Limited mechanical strength [128,129,130,131].
***Natural bioinks and their derivatives***	**Gel–MA**	Biocompatible [60], biodegradable, water soluble, and has the ability to encapsulate cells [90]. Promotes cell adhesion, spreading, viability, and proliferation [132]. Good printability, printing resolution, shape fidelity, and matrix for vascularization. Good mechanical properties at high concentrations.	Poor mechanical properties [90] at low concentrations. Reduced porosity and diffusion of nutrient and oxygen at high concentration.
	**HA–MA**	Increases viscosity [133] and has good shear-thinning properties [87] (depending on the molecular weight). Great tunability for special uses at different methacrylate degrees.	The hydrogels lack intrinsic cell adhesion sites, but can be modified through a simple photopolymerization reaction with arginine–glycine–aspartate (RGD) peptides to enhance cell adhesion, extension, and proliferation [134].
(**b**)			
***Types***	**Bioinks**	**Advantages**	**Disadvantages**
***Natural bioinks*** ***(hybrid)***	**Alginate and gelatin**	Biocompatible and biodegradable [119].	1% alginate–gelatin constructs become very weak over time and lack a flat surface for accurate measurements [135]. The composite also produces constructs with suboptimal mechanical properties in the long term [138]. Decreasing the concentration of alginate reduces the shape fidelity for lower concentrations of bioink [136].
	**Alginate and Gel–MA**	Alginate can enhance the printability and mechanical strength of the hollow tubes [60]. Alginate shell supports and confines the Gel–MA core to allow for UV crosslinking. A low concentration of Gel–MA (<2%) was successful. The bioprinting approach involving low-stiffness Gel–MA interiors and low-concentration alginate shells promoted the spreading of the enclosed cells within relatively brief timeframes. High degree of control over the 3D microenvironments for encapsulated cells [86]. The combination can produce proper viscosity for 3D bioprinting [60].	While adjusting polymer concentrations and crosslinking parameters allows for customization of mechanical properties and durability, dense polymer networks resulting from these adjustments may compromise cell viability and function [137].
	**Gel–MA and gelatin**	Thermo-crosslinking and irreversible photo (UV light)-crosslinking, yield stable and continuous generation of hollow structures. Good proliferation of many cell types in the Gel–MA/gelatin hollow fibers [132].	Inconsistency in their gelation mechanisms which can prevent proper integration and stability between layers or coaxial constructs while printing.
	**Gel–MA and HA**	Biocompatible, biodegradable, mechanically stable, and water soluble [119].	One significant difference is the potential for differential crosslinking rates between the two components. Gel–MA uses photo-crosslinking upon exposure to UV light, whereas HA does not crosslink at all. This discrepancy may result in uneven mechanical properties and structural integrity within the printed construct, resulting in compromised functionality and stability. The crosslinking occurs mainly between Gel–MA, with HA merely intermingling within the matrix.
	**Gel–MA and HA–MA**	Excellent performance in cartilage regeneration [95]. Good adhesion characteristics [133] and cell viability.	Lateral integration was a problem, perhaps due to the early time point analysis [95].
(**c**)			
***Types***	**Bioinks**	**Advantages**	**Disadvantages**
***Synthetic bioinks***	**Poly (ethylene) glycol**	Biocompatible, mechanically tough, [142] and has sufficient viscosity for bioprinting with high fidelity [143,144,145].	PEG does not crosslink on its own. This material lacks cell-adhering moieties needed for spreading, motility, and proliferation of the encapsulated cells [100,142].
	**Pluronic F-127**	Thermosensitive gelation behaviour, biocompatible, and non-toxic [103]. Gives temporary support to printing bioinks either when used as a support bath or as a sacrificial ink in coaxial and triaxial bioprinting. Viscosity and shear-thinning nature allow for printing with our small coaxial needle [110].	Time-consuming and may not be suitable for manufacturing complex organs with intricate vascular or neural networks when used as a sacrificial ink [147]. Also, it lacks biological activities, including cell adhesion as a sacrificial ink [147] or bioink [148].
	**Poly (ethylene) glycol dimethacrylate**	Mechanically stable and used for cartilage applications. Uses photo-crosslinking [146].	Low cytocompatibility [146].
***Natural/synthetic bioinks (hybrid)***	**Alginate, Gel–MA, and PEG–TA**	Has desired rheological properties, printability, sufficient mechanical strength, and bioactivity of the resultant constructs [100]. Alginate allowed rapid ionic crosslinking and shape maintenance of the initially bioprinted perfusable tubes through codelivery of CaCl_2_ solution [139,140,141]. Morphologies of the constructs could be permanently fixed by subsequent photo-crosslinking of the Gel–MA and PEG–TA components [100].	Constructs comprising alginate, Gel–MA, and PEG–TA, when subjected to prolonged UV exposure (>30 s), had a stiffer hydrogel network which impeded cell proliferation, spreading, and potentially migration [100]. The compressive moduli of the constructs also notably decreased following a 21-day culture period, primarily attributed to the degradation of the Gel–MA component. Consequently, maintaining perfusability beyond the 21-day culture period poses challenges [100].

#### 3.2.2. Pre-Printing: Rheological Properties and Their Impact on Printability

Rheological properties play a major role in determining the printability of bioinks in coaxial and triaxial bioprinting. These properties were addressed in the 85 articles on coaxial and triaxial bioprinting that we reviewed. However, a detailed analysis of these properties across all 85 articles was beyond the scope of our study. Instead, we have summarized some of the key rheological properties that are relevant to the printability of bioinks in coaxial and triaxial bioprinting [149,150]. The ability of the bioink to be extruded through the printer’s nozzle and the control of its flow directly influence the resolution, shape fidelity, and cell viability of the printed construct. Some major rheological parameters are commonly used to assess the printability of hydrogel bioinks, as follows:

(a) ***Viscosity (η)****:* An important determinant of extrusion and flow behaviour. High viscosity is often desired at low shear rates to prevent the collapse of larger constructs, while low viscosity at high shear rates allows for smooth extrusion [72,151].
(b) ***Yield stress (σ)****:* The minimum stress required to initiate flow in the bioink. High yield stress may hinder cell incorporation and motor movement in the 3D bioprinter [152].
(c) ***Storage modulus (G′) and loss modulus (G″):*** These values provide insights into the solid-like or liquid-like behaviour of the bioink during printing.

*(i)*  ***Viscosity and shear rate***

Viscosity directly influences the extrusion process and structural integrity of the printed object. For successful extrusion, bioinks need to have high viscosity at low shear rates and low viscosity at high shear rates [152]. This behaviour is referred to as **shear-thinning**, which allows for efficient extrusion of bioinks through the nozzle and smooth printing on the print bed. Viscosity is influenced by various factors, such as the molecular weight of the polymer, concentration, and the inclusion of rheological modifiers [153].

*(ii)*  ***Shear-thinning behaviour***

Shear-thinning behaviour occurs when the bioink exhibits high viscosity at low shear rates and low viscosity at high shear rates [154]. This behaviour is essential for bioprinting, as it ensures that the bioink flows easily through the nozzle during printing but maintains its shape fidelity once extruded. The **power-law model** [155,156] is commonly used to characterize shear-thinning behaviour mathematically, as follows:

(1) $$\eta =K\gamma^{n-1}$$

where *η* is the viscosity of the fluid at a particular shear rate, *K* is the flow consistency index (especially when the viscosity at a shear rate is 1 s^−1^), γ is the shear rate, and n is the shear-thinning index (also called the flow index of power-law index). When n = 1, the result is Newtonian; n < 1 indicates shear-thinning and n > 1 indicates shear-thickening. For n < 1, the material is shear-thinning, which is desirable in extrusion-based bioprinting.

*(iii)*  ***Yield Stress and Shear Recovery***

Yield stress represents the minimum force required to initiate flow in the bioink. High yield stress can impede cell incorporation and hinder the motion of the 3D bioprinter’s motor. **Gelatin** is notable for having low yield stress, which makes it a favorable material in certain bioprinting applications [152]. Additionally, shear recovery and oscillatory thixotropic measurements confirm the bioink’s stability during printing. The yield point, where the storage modulus (G′) becomes lower than the loss modulus (G″), is typically observed above 10 Pa or between 500–1000% strain [157].

To ensure successful bioprinting, the bioink must demonstrate specific rheological properties, such as suitable viscosity, shear-thinning behaviour, and low yield stress. Understanding these properties enables the optimization of hydrogel formulations for different printing technologies, such as coaxial and triaxial bioprinting, and ensures the production of high-quality, structurally stable 3D bioprinted constructs.

#### 3.2.3. Pre-Printing: Coaxial and Triaxial Nozzles

Coaxial and triaxial bioprinting technologies use multilayered nozzles to extrude bioink in a concentric fashion, which is fundamental for creating complex 3D constructs with distinct core and shell materials. The coaxial nozzle consists of two layers: an **outer shell (OS)** that extrudes the sheath material and an **inner core (IC)** that extrudes the core filament [32]. In contrast, the triaxial nozzle has three layers: an **outer shell (OS)** for the sheath, an **inner shell (IS)** for the middle filament, and an **inner core (IC)** for the central filament [59]. The dimensions of these nozzles are essential for determining the resolution of the printed structures and ensuring proper cell survival. For instance, coaxial nozzles typically have an **OS** diameter up to 7 mm and an **IC** diameter around 0.21 mm, producing finer-resolution fibers and curing properly. However, the nozzle diameter and length must be selected carefully, considering the specific application of the bioprinted construct [32].

Table 7 highlights the nozzle diameters reported in over 50 papers, which showed different sizes based on the application. Common diameters for the inner core (IC) range from 21 to 25 gauge (G) [31,34,107,118,136,157], as is optimal for cell survival. The inner shell (IS) often falls between 16 and 20 G [34,51,60,90], possibly to provide a balance between yielding proper support of the IC material and the bioink flow during extrusion. The outer shell (OS) typically ranges from 16 to 20 G [136,151,158,159,160], which ensures both mechanical stability and proper fitting of the IC.

*(i)*  *Core/Shell Diameter Ratio*

For a coaxial nozzle, the core-to-shell diameter ratio can be expressed as a function of the respective flow rates using the following equation [161]:

(2) $$\frac{Dc}{Ds}\sqrt{1-\sqrt{\left(\frac{Qs}{Qc+Qs}\right)}}$$

where

i.   Ds is the outer diameter of the shell;
ii.  Dc is the inner core diameter;
iii. Qs is the volumetric flow rate of the shell fluid;
iv. Qc is the volumetric flow rate of the core fluid.

This equation helps in optimizing the nozzle configuration based on the flow characteristics of both the core and shell fluids [161].

**Table 7:** Nozzle diameters used for coaxial and triaxial bioprinting (2013–2023).

Nozzle Components	Diameter Range (Gauge)	Most Commonly Used Range (Gauge)
Inner core (IC)	16–35 G	21–25 G
Inner shell (IS)	10–25 G	16–20 G
Outer shell (OS)	10–25 G	16–20 G

#### 3.2.4. Printing: Important Parameters

During coaxial and triaxial bioprinting, several key printing parameters are essential for successful printability and the fabrication of constructs. They include but are not limited to pressure, speed, layer height and temperature settings. These parameters play important roles in material deposition, resolution, and cell viability [162], as follows:

i.   Pressure: The printing pressure settings must be carefully adjusted based on various factors, like the material viscosity, nozzle size, and desired resolution. For triaxial bioprinting, this is even more complex due to multiple materials involved.
ii.  Speed: Printing speed is directly related to the time taken for the bioprinting process. The most commonly used printing speeds fall under 30 mm/s, which is typical for many studies [31,32,63,64].
iii. Cartridge and bed temperature: Temperature settings are crucial, especially for cell-laden hydrogels, as they can significantly impact cell survival. In particular, print cartridge temperature should be optimized for maintaining cell viability. The use of thermo-responsive materials may also require adjustments to the print bed temperature. For instance, Pluronic F-127 was used as a sacrificial ink during the printing of cell-laden core filaments. The print bed temperature was optimized to 30 °C to temporarily support the core material during the creation of the shell–core filament [31,33]. Despite this, many studies do not report temperature details, suggesting a need for further optimization and exploration in future research.

In short, for successful bioprinting, understanding and optimizing these printing parameters, including pressure, speed, and temperature, are essential for improving resolution, extrudability, and structural integrity of the final printed constructs.

#### 3.2.5. Post-Printing: Crosslinking Mechanisms

Crosslinking modifies the internal structure of a printed construct to achieve the desired mechanical properties. In the context of coaxial and triaxial bioprinting, this can be achieved through chemical, enzymatic, or physical mechanisms. The hydrogel composition influences which crosslinking method is most suitable. Crosslinking is a crucial step in bioprinting and can be applied during or after the printing process [12,31,32,64]. It is important to carefully optimize factors, like the concentration of crosslinking agents (chemical or UV-based), exposure time, and intensity to ensure good cellular viability and maintain the desired structural properties of the printed construct [31,32,33,65,95].

#### 3.2.6. Post-Printing: Mechanical and Cellular Tests

In coaxial and triaxial bioprinting, mechanical tests are essential for assessing the strength and stiffness of bioprinted constructs to ensure they meet the required tissue engineering standards. Common tests include compressive and tensile tests, which help evaluate how well the material withstands forces. For example, Kim & Kim [163] tested the mechanical properties of a core (PCL)–shell (collagen/alginate) structure, while Li et al. [112] examined the compressive strength of Alg/SF scaffolds. Both studies found that the core/shell structures and scaffolds outperformed controls in terms of strength and modulus. Stress/strain curves and Young’s modulus were also used to assess the materials’ mechanical properties. Swelling and degradation studies [64] are essential for evaluating the stability of the printed hydrogels. The swelling ratio measures how much the material expands when exposed to water, while the degradation study tracks the material’s breakdown over time under different conditions. Both tests provide insights into the hydrogel’s mechanical integrity and long-term viability.

Cellular tests, on the other hand, are designed to assess cell viability, metabolic activity, and other key factors within the printed constructs. These tests help evaluate how well the cells survive, proliferate, and function in the bioprinted environment. Viability is often calculated as the ratio of live cells to total cells. For example, Banigo et al. [31,33] evaluated the cell viability of their coaxially printed core filaments. Their results showed that more than 90% of the cells remained viable immediately after printing on Day 0, demonstrating the effectiveness of the printing process. These tests, along with others that can analyze gene expression and protein presence, help researchers understand how cells behave in the bioprinted structures and their potential for successful tissue engineering.

### 3.3. Applications of Coaxial and Triaxial Bioprinting

Bioprinting has emerged as a transformative technology in tissue engineering and regenerative medicine, offering great opportunities for the design and production of complex, three-dimensional (3D) constructs with precise control over cellular organization and the microenvironment. Among the various bioprinting techniques, coaxial and triaxial bioprinting have gained significant attention due to their ability to create hierarchical structures, incorporate multiple cell types, and mimic native tissue architecture. These techniques utilize coaxial or triaxial nozzles to deposit multiple bioinks and improve printing resolution compared to monoaxial nozzle bioprinting. In addition, they allow the fabrication of constructs in a single step, including the deposition of sacrificial bioink layers, thus enabling the production of constructs with intricate features and functionalities. A distinguishing feature of coaxial and triaxial bioprinting is their capability to create vascularized constructs, setting them apart from other extrusion processes. These characteristics make coaxial and triaxial bioprinting attractive methods for designing and developing various applications.

In this context, the major applications of coaxial and triaxial bioprinting include vascularization [34,35,58,59,60,64,81,90,100,102,111,115,116,120,122,137,160,164,165,166,167,168], cartilage tissue engineering [91,151,169], bone tissue engineering, renal tissue engineering [51], skeletal muscle tissue engineering [121,170,171,172,173], spinal cord tissue engineering [174], salivary tissue engineering [175], wound healing [120,171,172], 3D models [53,93,110,117,132,157,173,174,175,176,177], co-culture models [74,84,178], 3D constructs/scaffolds [57,63,92,136,137,179,180,181,182,183], soft or hard tissues, and clinical organ repair [55,77,86,107,112,163,184,185,186,187,188] (Table 8a–d and Table 9).

#### 3.3.1. Vascularized Tissue Printing

Vascularized tissue printing remains a significant challenge in tissue engineering due to the need for efficient methods to create fine nutrient delivery channels within cell-laden structures and to vascularize these channels effectively. To address this challenge, various bioprinting approaches have been explored, including coaxial and triaxial bioprinting, which offer promise for creating vascularized tissue constructs with physiologically relevant architectures.

Zhang et al. introduced a novel approach integrating coaxially bioprinted vascular microfluidic channels into bulk hydrogels for thick tissue and organ fabrication [82]. Here, the experimental setup comprises a single-arm robotic printer equipped with a pressure-regulated syringe pump for controlled deposition of biomaterials through a coaxial nozzle assembly. Sodium alginate, a commonly used hydrogel in tissue engineering, is dispensed through the sheath section of the coaxial nozzle, while calcium chloride solution is dispensed through the core section, initiating crosslinking to form tubular gels with hollow channels. Different coaxial nozzle assemblies with varying inner and outer needle diameters are utilized to assess the effect of dispensing parameters on microfluidic channel wall thickness, with measurements conducted using a digital microscope [82]. The approach permits the precise production of complex 3D microfluidic networks with controllable dimensions and mechanical properties through a layer-by-layer additive process, removing the need for post-processing and reducing the biomaterial volume by up to 21% compared to conventional scaffolding methods. Additionally, the integration of microfluidic channels provides mechanical support to the cellular assembly and enables controlled fluid transport, facilitating nutrient and waste exchange within tissue constructs, and holds promise for seeding co-culture cells and developing heterogeneous organ and tissue structures in the future [82]. Subsequent studies by Shao et al. [189] demonstrated the direct fabrication of vascularized nutrient delivery channels using coaxial bioprinting. By employing Gel–MA-based shell bioinks containing tissue-specific cells and surrounding HUVECs-laden gelatin core bioinks, bioprinted constructs with shell–core fibers and heterogeneous structures with vascular channels were achieved. Vascularization of these constructs was facilitated by dissolving the core gelatin fibers by increasing the temperature, enabling the deposition and adherence of HUVECs to the channel walls. This method allowed the development of tissue constructs with significant length and culture periods, demonstrating its potential for applications in tissue engineering, organ repair, and regenerative medicine. In the quest for scalable vascularized tissue constructs, the selection of appropriate bioinks is crucial.

Li et al. [112] described a hybrid bioink composed of C3A liver cancer cells mixed with alginate and silk fibroin (Alg/SF) as the sheath, surrounding a core sacrificial bioink consisting of a blend of Pluronic F-127 and calcium ions. This bioink combination facilitated the formation of a built-in vascular network with a regular structure and smooth microporous wall, offering desirable rheological properties and high cell viability and growth. Turner et al. [120] developed pre-vascularized scaffolds for wound care using core–shell bioprinting and a specialized cell-responsive composite bioink. This innovative approach facilitated stable construct formation and supported cell viability, proliferation, and early-stage vascular network development, leading to accelerated wound healing rates. Additionally, this method has potential applications for large-scale fabrication of pre-vascularized tissues, circumventing the need for complex microfabrication techniques. In another study, Hong et al. [64] demonstrated the one-step bioprinting of vascularized tissue constructs using a cell-laden gelatin bioink in a coaxial configuration. By encapsulating human dermal fibroblasts and human umbilical vein endothelial cells within distinct layers, they successfully created perfusable vascular constructs capable of maintaining cell viability and functionality for an extended period in vitro. Furthermore, coaxial bioprinting has shown promise in fabricating multilayer tubular constructs with heterocellular composition, resembling the anatomy of blood vessels and other tubular tissues. Pi et al. [60] demonstrated the fabrication of perfusable constructs using a custom bioink combination of Gel–MA, alginate, and eight-arm poly (ethylene) glycol acrylate with a tripentaerythritol core. These constructs exhibited high cell viability and growth, enabling the continuous perfusion of fluids and the interaction of different cell types in the fabricated layers.

On a more advanced level, Bosch-Rue et al. [58] demonstrated a methodology facilitating the rapid development of tissue-engineered blood vessel-like structures (TEBV), characterized by elevated cell viability and conducive conditions for cellular proliferation and natural alignment of vascular cells. Moreover, Wu et al. [93] delved into the fabrication of a three-dimensional (3D)-printed model mimicking aspects of the human bone marrow, aiming to foster the growth and proliferation of encapsulated multiple myeloma cells. This innovative approach provides valuable insights for modelling multiple myeloma, advancing drug development, and tailoring personalized therapeutic strategies in the realm of hematologic malignancies. Also, Zhang et al. [35] investigated a novel 3D bioprinting approach employing a triaxial nozzle to fabricate extensive vascularized tissue constructs. Subsequently, both channel-containing and channel-lacking constructs were produced and meticulously assessed. The results indicated comparable degradation profiles between constructs with and without channels, albeit with a lower compressive modulus and higher swelling rate observed in channel-containing constructs.

Biological scrutiny revealed enhanced outcomes in constructs featuring channels, including improved cell survival, proliferation, diffusion, migration, and the formation of vascular networks. In essence, this study showcased the feasibility of employing triaxial printing to fabricate large-scale vascularized tissue constructs, capable of precisely encapsulating diverse cell types while simultaneously establishing nutrient channels. This advancement holds promise for the preparation of expansive vascularized constructs for various applications [35].

A review by Kim and Cho [190] explored significant advances in 3D vascular bioprinting techniques, particularly in the context of disease model fabrication. One significant application is the use of coaxial bioprinting to model secondary hyperoxaluria, a condition affecting the kidney and intestine. This technique is particularly focused on replicating crucial functions, such as glucose reabsorption and the flow of tubular fluids.

In another study, Shan et al. [191] employed the Schiff base reaction mechanism in combination with coaxial 3D printing to crosslink gelatin molecules with an oxidized polysaccharide. This technique resulted in the creation of stable, slower-degrading gelatin and gelatin–alginate hydrogels. By integrating HUVECs within these oxidized pullulan (OxP)-crosslinked hydrogels, the team successfully bioprinted a continuous vascular network, a crucial step toward building cancer tissue models. These vascularized hydrogels are paving the way for the automated in vitro fabrication of complex bioartificial organs. Moving forward, further advancements in printing precision and vascular network optimization are expected to enhance their functionality and clinical applicability [191]. An interesting technique, known as coaxial sacrificial writing into functional tissues (co-SWIFT), has also emerged [192]. This technique enables the creation of hierarchically branched, multilayered vascular networks within dense cellular matrices and granular hydrogels. By using custom coaxial printheads, researchers were able to create robust connections between branching vessels, creating perfusable lumens surrounded by smooth muscle cell-laden shells. These vascularized tissues were successfully printed into matrices of various bioinks, including alginate microparticles and cardiac spheroids derived from human induced pluripotent stem cells (iPSCs). The endothelialized vessels presented excellent barrier function, while the perfused cardiac tissues matured, synchronized, and responded to cardioactive drugs in vitro. This offers great promise for scalable biomanufacturing, with applications in drug testing, disease modeling, and regenerative medicine [192]. In a systematic analysis of coaxial extrusion-based bioprinting, Sun et al. [193] combined computational fluid dynamics (CFD) with experimental evaluations to refine the bioprinting process. They utilized a pre-crosslinked alginate-collagen bioink and gelatin as a sacrificial material to form a double-layered hollow scaffold with human dermal fibroblasts (HDFs) and HUVECs encapsulated inside. The research team focused on modeling the bioink’s rheological behaviour within the coaxial nozzle, optimizing printing parameters, and developing evaluation criteria that included cell viability, print fidelity, and core–shell dimensions. Their work not only enhanced print quality but also reduced material costs and printing time, significantly enhancing efficiency in bioengineering applications [193]. These studies represent impressive progress in 3D vascular bioprinting and its potential to revolutionize tissue engineering. As techniques improve, the ability to fabricate complex, functional tissues for clinical and research purposes becomes increasingly attainable.

Overall, coaxial and triaxial bioprinting hold significant potential for the fabrication of vascularized tissue constructs for various applications in tissue engineering, organ repair, and regenerative medicine. By overcoming key challenges in vascularization and bioink selection, these approaches offer promising avenues for the development of functional and physiologically relevant tissues.

#### 3.3.2. Other Applications, Including Cartilage Tissue Engineering

Hydrogel-based scaffolds and decellularized extracellular matrix (dECM) have shown promise in tissue engineering, but their efficacy in treating volumetric muscle losses (VMLs) has been limited. Choi et al. [168] addressed this challenge by bioprinting volumetric muscle constructs using a coaxial nozzle system.

Their approach, which incorporated skeletal muscle dECM-based bioink and a granule-based printing reservoir, induced muscle fiber generation, innervation, and vascularization, resulting in significant functional recovery in a rat model of VML. These findings highlight the potential of bioprinted constructs for pre-clinical studies and the development of human-scale muscle tissues for various applications, including drug development and toxicity assessment [12]. Furthermore, coaxial bioprinting has been utilized to repair osteochondral defects. Di Bella et al. [95] employed a biopen system for intraoperative bioprinting of allogeneic adipose-derived mesenchymal stem cells encapsulated in a hyaluronic acid-based bioink, resulting in early cartilage regeneration in a sheep model.

Similarly, Hu et al. [194] and Kosik-Kozioł et al. [195] demonstrated the fabrication of multi-material constructs containing endothelial cells and bone marrow-derived mesenchymal stem cells for osteochondral repair. These studies showcase the potential of coaxial bioprinting for personalized treatments of musculoskeletal conditions.

Despite the advancements in renal tissue engineering, creating perfusable renal tubular tissue with clinically relevant cellular diversity remains challenging. Sing et al. [51] utilized coaxial 3D cell-printing to generate microfluidic hollow tubes containing renal tubular epithelial and endothelial cells, mimicking tubular/vascular renal parenchyma [51]. A novel hybrid bioink was produced to optimize cell functionality and maintain predefined hollow tubular structures, enabling the fabrication of complex tubes with tunable monolayer and bilayer features [51]. The approach was explored to prototype a vascularized renal proximal tubule-on-a-chip, demonstrating its potential for novel microfluidic renal tissue models and regenerative medicine applications, as evidenced by long-term graft survival and therapeutic efficacy in an in vivo renal disease model [51].

Also, the recent lack of effective therapy for salivary gland dysfunction, such as xerostomia resulting from radiation therapy, underscores the necessity for tissue engineering solutions. This involved isolating healthy salivary gland tissues pre-therapy, expanding cells in vitro, and creating functional neoglands for post-therapy implantation [170]. Utilizing 3D bioprinting approaches, Yin et al. [170] developed a microfluidics-based platform capable of producing thin, biocompatible hydrogel structures resembling salivary gland epithelia [170]. These structures, including solid fibers and hollow tubes, exhibited precise control over dimensions and cell incorporation, offering a promising approach for salivary gland restoration at microscale levels [170].

Shao et al. [90] developed microfiber constructs resembling blood vessels by using coaxial and triaxial bioprinting. These constructs exhibited structural diversity, cytocompatibility, and mechanical tunability, holding promise for regenerative applications, such as muscle, nerve, and blood vessel regeneration. Additionally, Gao et al. [196] demonstrated the fabrication of blood vessel structures using a hybrid bioink composed of vascular-tissue-derived dECM, endothelial progenitor cells, and proangiogenic drugs, suggesting potential applications in treating ischemic disorders and vascular injuries. In summary, coaxial and triaxial bioprinting offer a versatile approach for producing complex engineered tissues in tissue engineering.

Table 8a provides the synopsis of various bioink combinations used in coaxial bioprinting for a spectrum of distinct applications [55,77,82,85,122,160,164,178,197]. Table 8b displays additional bioink combinations explored for customized constructs using coaxial bioprinting [53,86,121,181,182,186,198]. Table 8c further details diverse bioink blends applied for specialized constructs through coaxial bioprinting [65,90,98,132,137,184,189,199]. Table 8d presents more examples of bioink formulations utilized in coaxial bioprinting for tailored applications [60,64,95,102,118,120,166,179]. Lastly, Table 8 summarizes bioink combinations employed in triaxial bioprinting to create customized constructs for various purposes [35,60,90,100,115].

**Table 8:** (**a**) A synopsis of diverse combinations of bioinks employed to customize constructs using coaxial bioprinting for a spectrum of distinct applications [55,77,82,85,122,160,164,178,197]. (**b**) A synopsis of diverse combinations of bioinks employed to customize constructs using coaxial bioprinting for a spectrum of distinct applications [53,86,121,181,182,186,198]. (**c**) A synopsis of diverse combinations of bioinks employed to customize constructs using coaxial bioprinting for a spectrum of distinct applications [65,90,98,132,137,184,189,199]. (**d**) A synopsis of diverse combinations of bioinks employed to customize constructs using coaxial bioprinting for a spectrum of distinct applications [60,64,95,102,118,120,166,179].

(**a**)					
***Outer Shell Bioink or Crosslinker***	**Inner Core Bioink or Crosslinker**	**Bath**	**Cell Type Used**	**Applications**	**References**
Alginate	Calcium chloride		**Outer shell**: bovine cartilage progenitor cells.	Direct fabrication of semi-permeable microfluidic channels for optimal cell cultivation and functionality.	Zhang et al. [82]
Alginate	Cell suspension	Calcium chloride	**Inner core**: human bone marrow mesenchymal stem cells.	High-yield production of mesenchymal stem cell (MSC)-derived extracellular vesicles (EVs) using microfiber culture.	Chen et al. [197]
Alginate	Cell suspension	Calcium chloride	**Inner core**: human bone marrow-derived mesenchymal stem cells and human ReNcell VM neural progenitor cells.	Investigating neural cell integration in 3D bioprinted bone constructs.	Zhang et al. [164]
Alginate	Cell suspension	Calcium chloride	**Outer shell**: glioma stem cells (GSC23) **Inner core**: glioma cell line (U118).	Microfiber constructs for drug development and screening.	Wang et al. [85]
Ultraviolet (UV)-sterilized sodium alginate	Calcium chloride		**Outer shell**: bovine cartilage progenitor cells.	Direct fabrication of cell-laden tubular channels to mimic the natural vascular system.	Yu et al. [77]
Alginate	Calcium chloride		**Outer shell**: L929 mouse fibroblasts.	Facile fabrication of hollow calcium alginate filaments using a coaxial nozzle, in which high strength cell-laden 3D structures with built-in microchannels can be produced.	Gao et al. [55]
Alginate	Calcium chloride mist		**Outer shell**: Neuro-2a cells (mouse neuroblasts).	Development of a mist-based printhead for droplet-based bioprinting of ionically crosslinking hydrogel bioinks.	Badr et al. [160]
Alginate	Calcium chloride		**Outer shell**: human umbilical vein smooth muscle cells.	Bioprinting of vascular conduits using a coaxial deposition system.	Zhang et al. [122]
Alginate	Gel–MA	Calcium chloride	**Outer shell**: adipose-derived mesenchymal stem cells and bone marrow mesenchymal stem cells. **Inner core**: human glioblastoma cell line (U87MG).	Coaxial microfiber platform for studying glioma–mesenchymal stem cell interactions. Coaxial extrusion bioprinted shell—MSC/core—U87MG microfiber is an ideal platform for tumor and stromal cell co-culture to observe tumor biological behaviour in vitro.	Jin et al. [178]
(**b**)					
***Outer Shell Bioink or Crosslinker***	**Inner Core Bioink or Crosslinker**	**Bath**	**Cell Type Used**	**Applications**	**References**
Calcium chloride	Sodium alginate and photocurable PEG–fibrinogen (PF)		**Inner core**: muscle precursor cells (C2C12), fibroblasts (BALB/3T3).	Fabrication of artificial skeletal muscle tissue with functional morphologies using an innovative 3D bioprinting approach.	Costantini et al. [121]
Alginate	Calcium chloride		**Outer shell**: L929 mouse fibroblasts, mouse vascular smooth muscle cells (MOVAS).	Fabrication of 3D hydrogel-based vascular structures with multilevel fluidic channels (macro-channel for mechanical stimulation and micro-channel for nutrient delivery and chemical stimulation).	Gao et al. [198]
Alginate	Calcium chloride and agarose solution		**Inner core**: rat myocardial cell lines (H9C2, ATCC).	Coaxial nozzle-assisted electrohydrodynamic printing of living 3D cell-laden constructs.	Liang et al. [181]
Alginate	hCB-CD34+ cells and BMSCs	Calcium chloride	**Inner core**: human cord blood-derived CD34+ cells (hCB-CD34+) and human bone marrow-derived mesenchymal stromal cells (BMSCs).	Mimicking the bone marrow niche using coaxial bioprinted scaffolds.	Chen et al. [182]
Alginate	hCB-CD34+ cells	Calcium chloride	**Outer shell**: human bone marrow-derived mesenchymal stromal cells (BMSCs). **Inner core**: human cord blood-derived CD34+ cells (hCB-CD34+).	Mimicking the bone marrow niche using coaxial bioprinted scaffolds.	Chen et al. [182]
Alginate	Gel–MA and calcium chloride		**Inner core**: human umbilical Vein endothelial cells (HUVECs).	Fabrication of cell-laden constructs with tunable microenvironments.	Liu et al. [86]
Alginate	Gel–MA and calcium chloride		**Inner core**: GFP- human umbilical vein endothelial cells (HUVECs).	Organ repair and tissue engineering.	Shao et al. [186]
Calcium chloride	Gel–MA and alginate		**Inner core**: human umbilical vein endothelial cells (HUVECs).	Development of biomimetic heterogeneous in vitro tissue models.	Colosi et al. [53]
(**c**)					
***Outer Shell Bioink or Crosslinker***	**Inner Core Bioink or Crosslinker**	**Bath**	**Cell Type Used**	**Applications**	**References**
Calcium alginate	Gel–MA	Calcium chloride	**Inner core**: human umbilical vein endothelial cells (HUVECs).	Fabrication of morphology-controlled microfibers.	Shao et al. [90]
Gel–MA	Alginate	Calcium chloride	**Outer shell**: endothelial progenitor cells (EPCs). **Inner core**: pancreatic islets.	Islet transplantation for type I diabetes treatment.	Liu et al. [137]
Gelatin and calcium chloride	Alginate and gelatin		**Outer shell**: human hepatocellular carcinoma cell line (HepG2) (monoculture) or human umbilical vein endothelial cell (HUVEC)–telomerase reverse transcriptase (TERT2) gene (co-culture). **Inner core**: HepG2 (co-culture) or HepG2 and fibroblast.	Pre-vascularization enhances in vitro cultivation of bioprinted grafts.	Bottcher et al. [199]
Gelatin	Gel–MA		**Outer shell**: human primary keratinocytes (HKCs). **Inner core**: immortalized cell line of human colorectal adenocarcinoma cells (Caco-2), spheroid-shaped human dermal papilla cells (HDPCs), and human fibroblasts (hFbs).	Vertical embedded extrusion bioprinting for tissue reconstruction.	Lian et al. [184]
Gel–MA	Gelatin		**Outer shell**: human umbilical vein endothelial cells (HUVECs), MC3T3-E1, or MDA-MB-231. **Inner core**: ECs.	Large-scale vascularized tissue constructs.	Shao et al. [189]
Gelatin/Gel–MA	PVA		**Outer shell**: Human umbilical vein endothelial cells (HUVECs).	Fabrication of hollow gelatin-based structures for tissue engineering and regenerative medicine.	Wang et al. [132]
HA–MA/Gel–MA	HA–MA/Gel–MA		**Inner core**: human infrapatellar fat pad-derived adipose Stem Cells (IPFP) [primary].	Handheld biofabrication tool (biopen) for tissue regeneration.	O’Çonnell et al. [98]
Gel–MA	HA–MA		**Inner core**: adipose-derived mesenchymal stem/stromal cells (ADSCs).	In situ surgical cartilage engineering.	Duchi et al. [65]
(**d**)					
***Outer Shell Bioink or Crosslinker***	**Inner Core Bioink or Crosslinker**	**Bath**	**Cell Type Used**	**Applications**	**References**
Gel–MA	HA–MA		**Inner core**: allogenic adipose mesenchymal stem cells.	Repair and regeneration of cartilage defects.	DiBella et al. [95]
Calcium chloride	Alginate and Gel–MA		**Inner core**: murine myoblast cell line C2C12 (ATCC).	Enhanced cellular organization in tissue-engineered scaffolds using multicompartmental hydrogel fibers.	Samandari et al. [166]
Gel–MA, alginate, and eight-arm poly (ethylene) glycol acrylate with a tripentaerythritol core	Calcium chloride		**Outer shell**: Various cell types.	Fabrication and creation of human cannular tissues.	Pi et al. [60]
Gel–PEG–TA (GPT) prepolymer	Gelatin		**Outer shell**: human dermal fibroblasts (HDFs). **Inner core**: human umbilical vein endothelial cells (HUVECs).	Coaxial nozzle-based bioprinting for vascular structure generation in a single step. In vitro disease modelling [12].	Hong et al. [64]
Polycaprolactone	Alginic acid with sodium salt	Calcium carbonate	**Inner core**: L929 mouse fibroblasts	Coaxial melt extrusion bioprinting of scaffolds for tissue engineering.	Cornock et al. [179]
Cell suspension containing fibrinogen as the core (CoF)	Sodium alginate, gelatin, and thrombin	Calcium chloride	**Inner Core**: red fluorescence protein (RFP)-expressing glioma stem cells (GSCs) and green fluorescence protein (GFP)-expressing mesenchymal stem cells (MSCs).	Fabrication of multicellular heterogeneous (brain) tumor fibers with the use of a custom-made coaxial extrusion 3D bioprinting system.	Dai et al. [118]
Gel–MA	Peptide-functionalized, succinylated chitosan (C)/dextran aldehyde (D)		**Outer shell**: human bone marrow-derived mesenchymal stem cells (hBMSCs). **Inner core**: human umbilical vein endothelial cells (HUVECs).	Wound healing applications.	Turner et al. [120]
Alginate, alginate lyase, Gel–MA, and PEG–DA 750	Pluronic F-127	Calcium chloride	**Outer shell**: vascular smooth muscle cells (VSMCs). **Inner core**: vascular endothelial cells (VECs) mixed with a growth medium containing 0.5% after 2 h (from printing).	Fabrication of small-diameter blood vessels with biomimetic cell layers.	Zhou et al. [102]

**Table 9:** A synopsis of diverse combinations of bioinks employed to customize constructs using triaxial bioprinting for a spectrum of distinct applications [35,60,90,100,115].

*Outer Shell Bioink or Crosslinker*	*Inner Shell Bioink or Crosslinker*	*Inner Core Bioink or Crosslinker*	*Bath*	*Cell Type Used*	*Applications*	*References*
Sodium alginate	Gel–MA	Gel–MA	Calcium chloride	**Inner core**: human umbilical vein endothelial cells (HUVECs)	Fabrication of morphology-controlled microfibers for tissue engineering	Shao et al. [90]
Gel–MA, alginate, and PEGOA	Gel–MA, alginate, and PEGOA	Calcium chloride		**Outer shell**: skeletal myocytes (C2C12), human bladder smooth muscle cells (HBdSMCs), and human umbilical vein endothelial cells (HUVECs). **Inner Core**: fibroblasts (NIH/3T3), human urothelial cells (HUCs), and human smooth muscle cells (hSMCs)	Microfluidic bioprinting for circumferentially multilayered tubular tissues	Pi et al. [60]
Calcium chloride	Gel–MA, alginate, and 4-arm PEG–TA	Calcium chloride		**Inner shell**: human umbilical vein endothelial cells (HUVECs) and mesenchymal stem cells (MSCs)	Versatile 3D bioprinting for perfusable vascular structures	Jia et al. [100]
Sodium alginate	Citrate buffer	Calcium chloride		**Outer shell**: mouse fibroblasts	Fabrication of engineered structures with branched micro-channels (ESBM) for vascularization	Li et al. [115]
Calcium chloride	Gel–MA and alginate	Gel–MA and alginate		**Outer shell**: human bone marrow mesenchymal stem cells **Inner shell**: human umbilical vein endothelial Cells (HUVECs)	Construction of large-scale vascularized tissue using triaxial bioprinting	Zhang et al. [35]

## 4. Conclusions and Future Perspectives

The advancements in 3D bioprinting, particularly in coaxial and triaxial bioprinting, have emerged from the dire need to develop an approach for rapidly and efficiently fabricating vascularized tissues. These tissues not only hold promise for tissue engineering and regenerative medicine but also offer significant potential for disease modelling and drug screening. The collaborative efforts of researchers from diverse disciplines have led to the evolution of 3D bioprinting. Recent progress in mechatronics, aimed at creating robust and efficient bioprinting machines, along with advancements in cell culture and biomaterial science, enabling the utilization of various bioink combinations for scaffold preparation, has propelled coaxial bioprinting as a preferred method for tissue construction. These bioprinting approaches facilitate the production of constructs in a single step, eliminating the need for additional post-extrusion processing. Depending on the type of nozzle employed, whether coaxial or triaxial, complex tissue constructs with varying properties can be generated. However, as emerging fields, further studies are required to address existing limitations. The potential for in situ crosslinking improves construct resolution, thereby enabling cell-cell and cell-material interactions. An exciting aspect of both coaxial and triaxial bioprinting lies in the development of biomimetic scaffold materials. Moreover, coaxial bioprinting enables the fabrication of drug- and biomolecule-loaded constructs, which could prove invaluable in various cell culture and drug screening analyses [12]. Coupled with advancements in automation and organoid bioprinting platforms, drug assays are anticipated to be significantly expedited.

**Figure 17:**
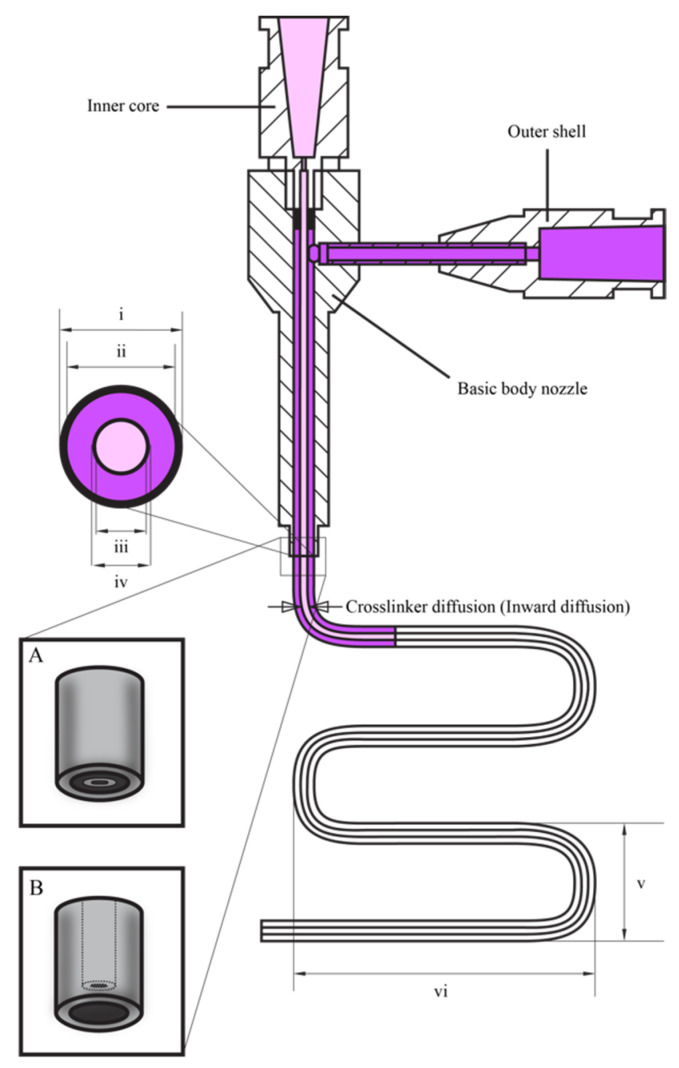
**Coaxial nozzle.** (**A**) Equal needle-length tips. (**B**) Shorter inner core needle length: (i) outer diameter of the outer shell (ii) inner diameter of the outer shell (iii) inner diameter of the inner core (iv) outer diameter of the inner core (v) arc of the filament section and (vi) length of the printed filament section.

For future advancements, the selection of hydrogel bioinks should be carefully considered based on the desired properties and specific application requirements. A key factor in this decision-making process is the bioprinting technique, which directly influences the quality of the printed constructs. Additionally, the choice of nozzle tip is critical for achieving the desired structural outcomes, such as filaments, tubes, and multilayered constructs. For instance, as shown in Figure 17A, we developed a nozzle with equal needle-lengths [31,33], expanding upon our idea to create a design capable of printing core filaments with good mechanical properties and good cell viability. This approach successfully balanced mechanical and cellular properties, resulting in good cell viability. This nozzle design could be further explored in the future for printing fibers with improved cellular response, enhanced printability, and better shape fidelity. In contrast, the nozzle tip shown in Figure 17B, with a shorter inner core length, was designed by us, drawing upon our idea to focus on optimizing shape fidelity and mechanical properties. When combined with cell-friendly materials, such as collagen or gelatin, this nozzle design could potentially produce fibers with a more favorable cellular response.

Moreover, nozzle tips tailored for printing hollow structures could offer significant benefits, particularly in terms of improving the overall mechanical integrity and cellular interaction within these constructs. For example, Milojevic et al. [63] designed a specialized nozzle with a unique tip to print complex structures, showing promise for future applications in tissue engineering.

**Figure 18:**
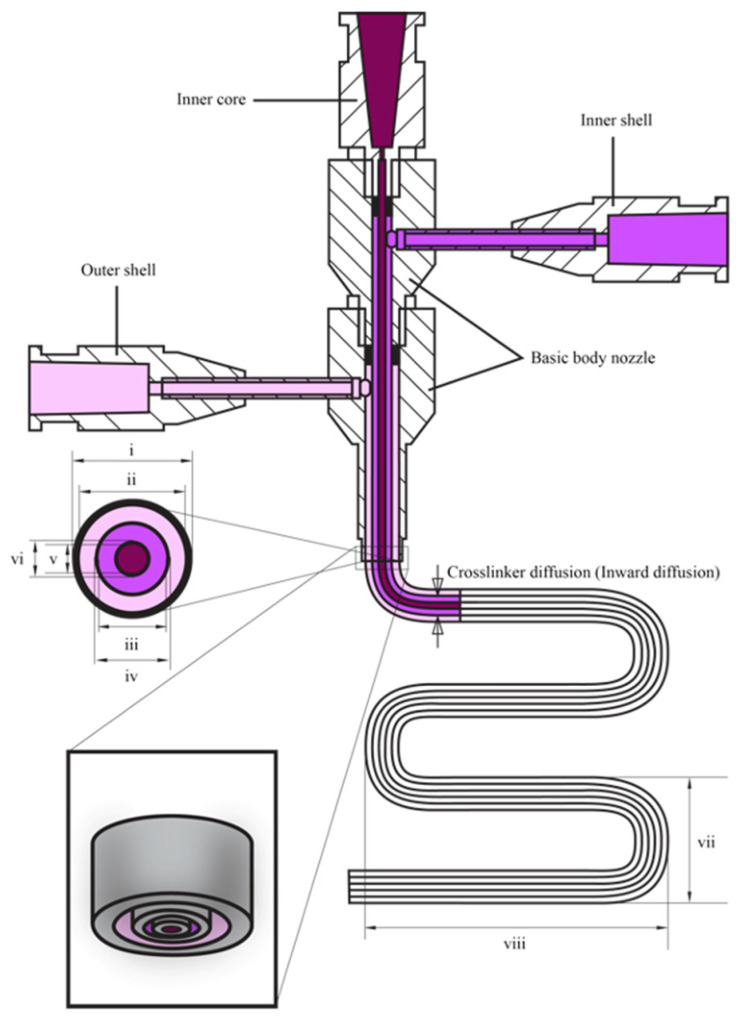
Triaxial nozzle. Equal needle-length tips: (i) outer diameter of the outer shell (ii) inner diameter of the outer shell (iii) inner diameter of the inner shell (iv) outer diameter of the inner shell (v) inner diameter of the inner core (vi) outer diameter of the inner core (vii) arc of the filament section and (viii) length of the printed filament section.

As seen in Figure 18, a triaxial nozzle with equal needle-length tips was designed by us, drawing upon our idea concerning the potential of multi-material printing, enabling the fabrication of complex, multilayered structures, such as scaffolds for tissue regeneration or vascular networks. Our unique nozzle design ideas can be further explored for coaxial and triaxial bioprinting to create complex fiber and tubular structures that support advanced tissue engineering applications. Utilizing these nozzle configurations could lead to more precise control over the spatial arrangement of cells and materials, improving the performance and functionality of printed constructs.

In addition, although still in its early stages, the integration of machine learning (ML) and computer-aided methods offers the potential to minimize processing errors or even biomaterial research and 3D cell culture to improve bioprinting techniques for tissue provide real-time solutions when issues occur. ML can also tap into existing datasets from engineering [200]. By integrating machine learning algorithms with 3D bioprinting, it is possible to identify more effective bioinks, optimize printing parameters, and detect defects during the printing process [201].

## Figures and Tables

**Table 1:** Comparison of the four most common bioprinting techniques [16,17,18,19,20,21].

*Properties*	*Laser-Based*	*Inkjet-Based*	*Stereolithography-Based*	*Extrusion-Based*
***Material***	High gelation speed [17]	Multicell printing is possible, critical for complex organs [17]	Photosensitive materials [18]	Broad variety of biocompatible materials [17]
***Material viscosities***	1–300 mPa∙s [16]	3.5–12 mPa∙s [21]	No limitation [18]	30 to >6 × 10^7^ mPa∙s [16]
***Crosslinking mechanism***	Chemical, Photo-crosslinking [16]	Chemical, photo-crosslinking [16]	Photo-crosslinking [19]	Chemical, photo-crosslinking, shear-thinning, temperature [21]
***Nozzle dynamics***	Nozzle free [20,21]	Non-contact nozzle, but nozzle can clog [21]	Nozzle free [20]	Shear stress induced by nozzle wall and extrusion pressure [21]
***Printer cost***	High [16,18,21]	Low [18]	Low [18]	Low [21]/medium [16]
***Resolution***	20–100 μm [20]	100–500 μm [20]	20–100 μm [20]	100–500 μm [20]
***Printing Speed***	Medium [20]	Fast [20]	Fast [20]	Slow [20]
***Cell density***	Medium [16,20]	Low [16,20]	Medium [20]	High [16,20,21]
***Cell viability***	<85% [16,21]	>85% [16,21]	25–90% [20]	40–80% [16,21]
***Advantages***	No nozzle, no clogging issues	Fast printing speed [18,19]	Fast printing speed [19]	Many cells and materials delivery
	Reduced problems of shear stress which may affect cell viability [18]	Low cost [18]	Reduced fabrication time [18]	Shape fidelity is high post-printing [18]
***Drawbacks***	High equipment cost [18,19]	Low viscosity needed [18,19]	Photosensitive materials	Slow print speed
	Heat from laser [18] could damage cells [18,19]	Low cell density needed [18,19]	Possible deoxyribonucleic acid (DNA) damage by ultraviolet (UV) light [18]	Smaller diameter nozzle can reduce cell viability [18]

## Data Availability

The original contributions presented in the study are included in the article, further inquiries can be directed to the corresponding author.

## Abbreviations

3D	Three-dimensional
ADSCs	Adipose-derived mesenchymal stem/stromal cells
Alg/SF	Alginate and silk fibroin
BALB/3T3	BALB/c mouse embryonic fibroblast cell line
ATCC	American type culture collection
BMSCs	Bone marrow-derived mesenchymal stromal cells
CaCl_2_	Calcium chloride
CFD	Computational fluid dynamics
C2C12	Mouse myoblast cell line
C3A	Human hepatocellular carcinoma cell line
co-SWIFT	Coaxial sacrificial writing into functional tissues
dECM	Decellularized matrix
DA	Diacrylate
DNA	Deoxyribonucleic acid
EBB	Extrusion-based bioprinting
ECM	Extracellular matrix
EPCs	Endothelial progenitor cells
ESBM	Engineered structures with branched micro-channels
EVs	Extracellular vesicles
Gel	Gelatin
Gel–MA	Gelatin–methacryloyl
Gel–TA	Gelatin–tyramine
Gel–PEG–TA	Gelatin-poly (ethylene) glycol–tyramine
GFP	Green fluorescence protein
GSC23	Glioma stem cells
HA	Hylauronic acid
HA–MA	Hyaluronic acid–methacrylate
HA–TA	Hyaluronic acid–tyramine
HBdSMCs	Human bladder smooth muscle cells
HDFs	Human dermal fibroblasts
HDPCs	Human dermal papilla cells
HepG2	Hepatocellular carcinoma cell line
HFbs	Human fibroblasts
HKCs	Human primary keratinocytes
HRP/H_2_O_2_	Horseradish peroxidase/hydrogen peroxide
hSMCs	Human smooth muscle cells
HUCs	Human urothelial cells
HUVECs	Human umbilical vein endothelial cells
H9C2 (ATCC)	Rat heart myoblast cell line
hCB-CD34+	Human cord blood CD34+ cells
IPFP	Human infrapatellar fat pad-derived adipose stem cells
iPSCs	Induced pluripotent stem cells
MC3T3-E1	Mouse calvarial osteoblast cell line
MDA-MB-231	Human breast cancer cell line
ML	Machine learning
MOVAS	Mouse vascular smooth muscle cells
MSCs	Mesenchymal stem cells
PEG	Poly (ethylene) glycol
PEG–DA	Polyethylene glycol–diacrylate
PEG–TA	Polyethylene glycol–tyramine
PCL	Poly (ε-caprolactone)
PEO	Polyethylene oxide
PF	Photocurable PEG–fibrinogen
PPO	Polypropylene oxide
PLA	Poly (lactic acid)
PRISMA	Preferred reporting items for systematic reviews and meta-analyses
PubMed:	Public medical literature database
OxP	Oxidized pullulan
RFP	Red fluorescence protein
RGD	Arginylglycylaspartic acid
TA	Tyramine
TE	Tissue engineering
TEBV	Tissue-engineered blood vessel-like structures
TERM	Tissue engineering and regenerative medicine
TERT2	Telomerase reverse transcriptase
U118	Glioma cell line
U87MG	Uppsala 87 malignant glioma, human glioblastoma cell
UV	Ultraviolet
VECs	Vascular endothelial cells
VM	Ventral mesencephalon
VML	Volumetric muscle losses
VSMCs	Vascular smooth muscle cells
Xan-CHO/NOCC	Aldehyde xanthan carboxymethyl chitosan
